# Screening and Genetic Network Analysis of Genes Involved in Freezing and Thawing Resistance in *DaMDHAR*—Expressing *Saccharomyces cerevisiae* Using Gene Expression Profiling

**DOI:** 10.3390/genes12020219

**Published:** 2021-02-03

**Authors:** Il-Sup Kim, Woong Choi, Jonghyeon Son, Jun Hyuck Lee, Hyoungseok Lee, Jungeun Lee, Seung Chul Shin, Han-Woo Kim

**Affiliations:** 1 Advanced Bio-Resource Research Center, Kyungpook National University, Daegu 41566, Korea; 92kis@hanmail.net; 2 Korea Polar Research Institute, Incheon 21990, Korea; woong@kopri.re.kr (W.C.); jonghyeon_son@kopri.re.kr (J.S.); junhyucklee@kopri.re.kr (J.H.L.); soulaid@kopri.re.kr (H.L.); jelee@kopri.re.kr (J.L.); ssc@kopri.re.kr (S.C.S.); 3 Department of Polar Science, University of Science and Technology, Incheon 21990, Korea

**Keywords:** Antarctic plant, *Deschampsia antarctica*, monodehydroascorbate reductase, freezing and thawing, gene expression profiling, transgenic yeast

## Abstract

The cryoprotection of cell activity is a key determinant in frozen-dough technology. Although several factors that contribute to freezing tolerance have been reported, the mechanism underlying the manner in which yeast cells respond to freezing and thawing (FT) stress is not well established. Therefore, the present study demonstrated the relationship between *DaMDHAR* encoding monodehydroascorbate reductase from Antarctic hairgrass *Deschampsia antarctica* and stress tolerance to repeated FT cycles (FT2) in transgenic yeast *Saccharomyces cerevisiae*. *DaMDHAR*-expressing yeast (DM) cells identified by immunoblotting analysis showed high tolerance to FT stress conditions, thereby causing lower damage for yeast cells than wild-type (WT) cells with empty vector alone. To detect FT2 tolerance-associated genes, 3′-quant RNA sequencing was employed using mRNA isolated from DM and WT cells exposed to FT (FT2) conditions. Approximately 332 genes showed ≥2-fold changes in DM cells and were classified into various groups according to their gene expression. The expressions of the changed genes were further confirmed using western blot analysis and biochemical assay. The upregulated expression of 197 genes was associated with pentose phosphate pathway, NADP metabolic process, metal ion homeostasis, sulfate assimilation, β-alanine metabolism, glycerol synthesis, and integral component of mitochondrial and plasma membrane (PM) in DM cells under FT2 stress, whereas the expression of the remaining 135 genes was partially related to protein processing, selenocompound metabolism, cell cycle arrest, oxidative phosphorylation, and α-glucoside transport under the same condition. With regard to transcription factors in DM cells, *MSN4* and *CIN5* were activated, but *MSN2* and *MGA1* were not. Regarding antioxidant systems and protein kinases in DM cells under FT stress, *CTT1*, *GTO*, *GEX1*, and *YOL024W* were upregulated, whereas *AIF1*, *COX2*, and *TRX3* were not. Gene activation represented by transcription factors and enzymatic antioxidants appears to be associated with FT2-stress tolerance in transgenic yeast cells. *RCK1*, *MET14*, and *SIP18*, but not *YPK2*, have been known to be involved in the protein kinase-mediated signalling pathway and glycogen synthesis. Moreover, *SPI18* and *HSP12* encoding hydrophilin in the PM were detected. Therefore, it was concluded that the genetic network via the change of gene expression levels of multiple genes contributing to the stabilization and functionality of the mitochondria and PM, not of a single gene, might be the crucial determinant for FT tolerance in *DaMDAHR*-expressing transgenic yeast. These findings provide a foundation for elucidating the *DaMDHAR*-dependent molecular mechanism of the complex functional resistance in the cellular response to FT stress.

## 1. Introduction

There are only two species of flowering plants in the Antarctic Peninsula, Antarctic hairgrass (*Deschampsia antarctica*) and Antarctic pearlwort (*Colobanthus quitensis*). During their entire life cycle, Antarctic plants are exposed to multiple abiotic stresses, including extreme temperatures, varying oxygen concentrations, water and nutrient deficiency, extremely short growing seasons, common summer frosts, strong winds, low light quality, and photoperiod changes [1,2,3,4]. Low temperatures, particularly freezing temperatures, can dramatically affect plants from the cellular level to ecosystem scales [5]. To overcome extreme temperature conditions, these Antarctic plant species have evolved a wide range of cell rescue systems involved in physiological and molecular functions for increasing their cold resistance through enhanced metabolic rate upon cold acclimation [4,6,7]. For example, plants in the Antarctic region have long life cycles, extended leaf and flower primordia development, well-developed root systems, and efficient photosynthetic and respiratory systems at ≤10 °C [2,6,8,9]. Various cellular changes induced by low-temperature lead to the excess accumulation of toxic compounds, particularly reactive oxygen species (ROS) [2,10]. The high accumulation of ROS generates oxidative stress [11]. ROS can induce DNA damage, protein oxidation, membrane lipid peroxidation and pigment destruction [2,6,12]. Therefore, a considerable amount of research has been conducted to explore the correlation between ROS scavenging and plant stress tolerance under extreme temperatures [13,14,15].

To neutralize ROS generated by cold stress, plants, including those in the Antarctic, have developed a wide range of cell rescue systems such as antioxidant systems [15,16,17]. One includes enzymatic components, such as superoxide dismutase, catalase (CAT), glutathione (GSH) peroxidase, and peroxiredoxins, and especially the ascorbate (AsA)–GSH cycle in plants [18,19]. The other component includes non-enzymatic antioxidants, including ascorbic acid (AsA; vitamin C), GSH, flavonoids, and polyphenol-derived compounds [20]. Among them, AsA, an abundant component of plants, plays an important role as a strong and active ROS scavenger [11]. Under oxidative stress, AsA directly participates in detoxifying excess ROS and leads to oxidized forms [21]. In this process, AsA is oxidized to monodehydroascorbate (MDHA), and MDHA is further oxidized to dehydroascorbate (DHA) via spontaneous reaction [19]. To enhance the antioxidative capacity and redox state of AsA after an increased AsA pool, oxidized AsA is required to reduce AsA via rapid, effective recycling mediated by DHA reductase (DHAR) and MDHA reductase (MDHAR) in the presence of GSH [11,22]. Several studies have reported that overexpression of either *DHAR* or *MDHAR* enhanced plant resistance against various abiotic stresses [23,24,25]. It is particularly suggested that the role of MDHAR in AsA regeneration is more efficient than that of DHAR under oxidative stress [21,26]. Therefore, the improvement of acquired tolerance to extreme temperatures is often related to enhanced activities of enzymes involved in the antioxidant systems of plants [11,27].

*Saccharomyces cerevisiae* is a eukaryotic unicellular budding yeast widely distributed in natural environments as well as found in association with various insects, animals, and plants [28,29]. *S*. *cerevisiae* plays an important role in food chains and nutrient cycles such as nitrogen and sulfur and is used to produce food, beverages, chemicals, pharmaceuticals, biocontrol agents, and industrial enzymes as well as in agriculture [30,31]. Moreover, *S*. *cerevisiae* is the main yeast responsible for biomass-based alcoholic fermentation using hydrolyzed glucose derived from rice, wheat, barley, corn, and grape in a commercially important plant-based agriculture field [32,33]. During fermentation, yeast cells are dynamically challenged by mixed and interrelated stressors, especially high ethanol concentration [34]. Increased ethanol concentration harms cells and affects the fermentation quality and yield [35]. Furthermore, ethanol production depends on yeast’s ability to adapt to sudden or continuous environmental changes, including disruption of physical (temperature, light, radiation, and pressure) and chemical (water and nutrient availability, acidity and pH, oxygen relations and presence of inhibitory and antimicrobial substances) factors, and ethanol stress during fermentation [36]. To solve these limitations, various strategies in yeast have been developed. The specific properties of yeast can be altered via genetic improvement through numerous approaches, including sexual breeding, parasexual hybridization, random mutagenesis, metabolically genetic engineering such as genome editing, and transformation using recombinant genes [37,38,39]. Using transgenic biotechnology, yeast is engineered to express foreign and exogenous genes to generate products of industrial interest, such as bioethanol-based biofuel and secondary metabolites [37,40,41]. Owing to their academic and industrial interests, studies on stress tolerance in yeast are of fundamental scientific importance [42]. Stress resistance can be achieved by developing stress-tolerant yeast strains via effective genetic engineering of relevant genes [37].

Among abiotic stresses, yeasts are frequently exposed to freezing–thawing (FT) cycles (a type of abiotic stress) and several cellular factors have been identified with yeast survival after FT stress [43]. FT technology, particularly freezing, is common in industrial applications; therefore, the freeze tolerance of the yeast used is of paramount importance [43]. The FT stress response is vital because of its integration with a larger stress response network that includes chilling, high temperatures and oxidative stress [42]. Screens for strains better suited for use in frozen doughs have been based on the few microbial freeze tolerance mechanisms identified to date [43]. Therefore, additional research is required for resolving the issues associated with frozen dough technology and developing new yeast strains better suited for application in this technology. Unlike in plants, information available about stress response involving *MDHAR* in yeast is limited. In the present study, a cDNA encoding *MDHAR* (*DaMDHAR*) from the Antarctic hairgrass *D*. *antarctica* (*DaMDHAR*) was cloned, and its function was analyzed in a genetically modified *S. cerevisiae* strain. Here, heterologous *DaMDHAR* expression improves tolerance to FT stress in *S. cerevisiae* by providing critical information to better understand FT tolerance mechanisms via genetic network analysis and characterizes its value as a genetic resource. Furthermore, these findings improve the understanding of the development of FT-tolerant yeast strains and demonstrate the potential application of transgenic yeast in the cold-based industrial field.

## 2. Materials and Methods

### 2.1. Establishment of DaMDHAR-Expressing Yeast

Total RNA was isolated from the leaves of *D*. *antarctica* plants using the RNeasy plant mini kit (Qiagen, Hilden, Germany). cDNA was synthesized by reverse transcription-polymerase chain reaction (RT–PCR). The *DaMDHAR* coding region was amplified from the cDNA by PCR using *Pfu* polymerases (Roche, Basel, Switzerland). The reaction conditions were as follows: initial denaturation at 94 °C for 3 min, followed by 30 cycles of 94 °C for 30 s, 58 °C for 30 s, and 72 °C for 1.5 min, and a final extension at 72 °C for 7 min. The sense and antisense primer set used for PCR cloning of the *DaMDHAR* gene are as follows: 5′-ATTCTAGAACTAGTGGATCC**ATGGCGACGGAGAAGCACTTC**-3′ and 5′-TCGACGGTATCGATAAGCTT**TCAGATCTTGCTGGCGAACAGG**-3′. The nucleotide sequences of the p426GPD vector corresponding to endonucleases (*BamH*I and *Hind*III) are underlined. The primer sequence involved in the *DaMDHAR* gene is boldfaced. The purified PCR product was cloned into the yeast expression vector p426GPD under the control of the *glyceraldehyde*-*3*-*phosphate dehydrogenase* (*GPD*) promoter (Euroscarf, Frankfurt, Germany) using the sequence- and ligation-independent cloning (SLIC) method with T4 DNA polymerase [44]. After sequence confirmation, the vector construct of p426GPD::DaMDHAR was transformed into *S. cerevisiae* BY4741 cells using the PEG/LiCl method [45]. Transformants were selected by plating cells on an SD agar medium (0.67% yeast nitrogen base without amino acids and with ammonium sulfate, 0.192% yeast synthetic dropout medium lacking uracil, 2% glucose, and 1.5% agar) at 28 °C for 3 days. A single colony was streaked, cultured under the same conditions, and then used for subsequent experiments. Yeast strains used in this study were represented in Table S1.

### 2.2. Stress Response Assay to FT

Yeast cells (initial concentration adjusted to 1 × 10^6^ cells/mL) grown overnight at 28 °C were inoculated into a fresh Yeast Extract Peptone Dextrose (YPD) broth medium (1% yeast extract, 2% peptone, and 2% dextrose) and cultured at 28 °C for 6 h with shaking (160 rpm). Early log-phase yeast cells (OD_600_ ≈ 1.0) were then challenged to FT condition. To induce FT stress, cells were frozen at −80 °C for 0.5 h and thawed out at 30 °C for 0.5 h, and the process was repeated six times. Stressed yeast cells were serially diluted 10-fold (10^0^–10^−4^) with YPD broth medium. Then, a 5 µL aliquot of each dilution was spotted onto the YPD agar plates (YPD plus 1.5% agar), incubated for 2–3 days at 28 °C, and photographed. For growth kinetics, yeast cells exposed to FT twice were resuspended into YPD broth medium and then monitored by measuring the optical density at 600 nm at 1 h interval for 12 h.

### 2.3. RNA Isolation

Total RNA was extracted from yeast cells challenged twice to FT stress using a TRIzol method according to the manufacturer’s instructions (Invitrogen/Thermo Fisher Scientific, Camarillo, CA, USA). Briefly, 100 mg yeast cells were mixed with an equal volume of glass beads (425–600 µm; Sigma-Aldrich/Merck, St. Louis, MO, USA) combined with TRIzol reagent (1 mL). After cell disruption, 0.2 mL chloroform was added, and the samples were mixed manually for 15 s and then incubated for 3 min. After centrifugation (10,000× *g*) for 15 min at 4 °C, the aqueous layer was recovered and mixed with 0.25 mL of 3 M sodium acetate (pH 5.2) and 0.25 mL isopropanol. The RNA pellet was obtained by centrifugation and washed twice with 75% ethanol. RNA quantity was measured using a NanoDrop spectrophotometer (Nano-Drop Technologies, Wilmington, DE, USA).

### 2.4. Gene Expression Profiling

For 3′-quant sequencing, library construction was performed using the QuantSeq 3′-mRNA-Seq library prep kit (Lexogen, Vienna, Austria) according to the manufacturer’s instructions. In brief, 500 ng each of total RNA was prepared, and an oligo-dT primer containing an Illumina-compatible sequence at its 5′ end was hybridized to the RNA. As a result, cDNA was synthesized by reverse transcription. After degradation of the RNA template, the second-strand synthesis of the cDNA was initiated by a random primer containing an Illumina-compatible linker sequence at its 5′ end. The double-stranded library was purified using magnetic beads to remove all reaction components. The library was amplified by adding the complete adapter sequences required for cluster generation. The finished library was purified from PCR components. High-throughput sequencing was performed as single-end 75 sequencings using NextSeq 500 (Illumina, San Diego, CA, USA). QuantSeq 3′-mRNA-Seq reads were aligned using Bowtie2 [46]. Bowtie2 indices were either generated from the genome assembly sequence or the representative transcript sequences for aligning to the genome and transcriptome [47]. The alignment file was used for assembling transcripts, estimating their abundances, and detecting differential gene expression. Differentially expressed genes were determined based on counts from unique and multiple alignments using coverage in BEDTools [48]. The RC data were processed through the quantile normalization method using EdgeR within R using Bioconductor (https://bioconductor.org/) [49]. Gene classification was based on searches done on DAVID (http://david.abcc.ncifcrf.gov/) and Medline (http://www.ncbi.nlm.nih.gov/) databases. The genetic network of the differentially expressed genes was framed using String (http://apps.cytoscape.org/apps/stringapp) and ClueGo (http://apps.cytoscape.org/apps/cluego) [50]. First, experimental data were integrated and analyzed using ClueGO App. of Cytoscape plug-ins and functional analysis was performed using gene ontology (GO). Next, a gene-gene network analysis using interaction data from the STRING database was imported into Cytoscape and visualized. Genes with a change of ≥2-fold and a log_2_-based normalized value of ≥ 8 (log_2_3) were defined as true positives; genes with an absolute log_2_ change value of ≤ 0.1-fold were fined as true negatives.

### 2.5. Western Blot Analysis

Yeast cells treated twice with FT stress were harvested by centrifugation at 2000× *g* for 3 min at 4 °C. The crude protein extracts were prepared using glass beads, as reported previously [41]. Cells were washed three times with cold phosphate-buffered saline (PBS; Invitrogen) and resuspended in lysis buffer containing 50 mM HEPES (pH 7.2), 5% glycerol, 10 mM 1,4-dithiothreitol, 1 mM phenylmethylsulfonyl fluoride, and protease inhibitor cocktail (Sigma-Aldrich) with an equal volume of glass beads (425–600 µm; Sigma-Aldrich). After vigorously vortexing four times for 1 min each at 2 min intervals on ice, the lysates were centrifuged at 13,000× *g* for 20 min at 4 °C, and the supernatants were used as protein extracts. The protein concentration was determined using protein dye reagent (Bio-Rad, Hercules, CA, USA). Bovine serum albumin was used as a standard. Total protein (20 μg) was resolved on sodium dodecyl sulfate-polyacrylamide gel electrophoresis and electrophoretically transferred to a polyvinylidene difluoride membrane. The membrane was incubated with a blocking buffer containing 5% non-fat skim milk in Tris-buffered saline containing 0.05% Tween-20 (TBST) plus 0.02% sodium azide for 1.5 h at 25 °C, and then incubated at 4 °C overnight with an anti-OsMDHAR antibody appropriately diluted in blocking buffer without sodium azide. The blot was washed four times every 10 min for 40 min with TBST and then incubated for 1.5 h at 25 °C with appropriately diluted goat anti-rabbit IgG-horseradish peroxidase secondary antibody (Santa Cruz Biotechnology Inc., Dallas, TX, USA). After washing four times every 10 min for 40 min with TBST, the bands were visualized using ECL Western blotting detection reagent (GE Healthcare, Little Chalfont, UK). The anti-tubulin antibody (Santa Cruz Biotechnology) was used as a loading control. The anti-OsMDHAR antibody produced in rabbits through the purified OsMDHAR protein was used instead of the anti-DaMDHAR antibody because the amino acid sequence of DaMDHAR has more than 90% homogeneity of that of OsMDHAR [51].

### 2.6. NADPH Assay

Yeast cells exposed to FT stress were harvested by centrifugation and resuspended in cold PBS solution. After vigorous vortexing with glass beads 4 times for 1 min each at 2-min intervals on ice, the lysates were centrifuged at 13,000× *g* for 20 min at 4 °C, and the cleared supernatants were used for NADPH assay. Intracellular concentrations of NADPH were quantified using the spectrophotometric quantification method, as previously reported [52]. The NADPH levels were expressed as nanomoles per milligram protein.

### 2.7. Statistical Analysis

Significant differences in the measured parameters were identified using Origin 2020. All experiments were performed at least three times independently. The results are expressed as the mean ± standard deviation. The results of the spotting assays are representatives of at least two independent experiments performed under identical conditions. A probability analysis to determine statistical significance was performed using the Student’s *t*-test with a two-tailed distribution.

## 3. Results

### 3.1. Generation of DaMDHAR-Expressing Transgenic Yeast and Their Stress Response to FT

**Figure 1:**
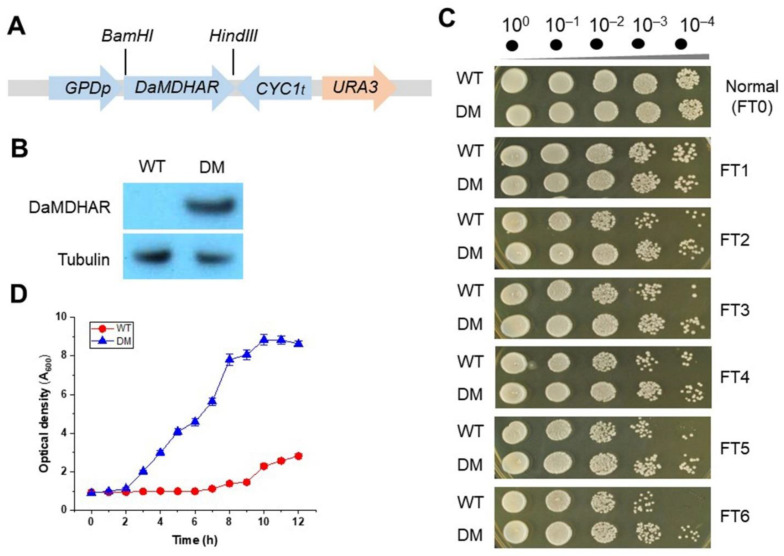
Cellular response in cDNA encoding *MDHAR* (*DaMDHAR*)-expressing transgenic yeast cells to freezing and thawing (FT) stress. (**A**) Schematic diagram of *DaMDHAR*-expression vector constructs for *S*. *cerevisiae*. *GPDp*, glyceraldehyde-3-phosphate dehydrogenase (GPD) promoter; *DaMDHAR*, the gene encoding MDHAR from Antarctic hairgrass *D*. *antarctica*; *CYC1t*, cytochrome c (CYC1) terminator; *URA3*, uracil gene as a selectable marker; *BamHI* and *HindIII*, restriction endonuclease. (**B**) *DaMDHAR* expression at the protein level was performed by immunoblotting assay. Tubulin protein was used as a loading control. (**C**) Stress-response assay under six-repeated FT cycles. Exponential yeast cells (A_600_ ≈ 1.5) were exposed to six-repeated FT cycles from FT1 to FT6 and then serially diluted to 10^–4^ with Yeast Extract Peptone Dextrose (YPD) broth. Five microliters of the diluted solution were spotted onto the YPD agar broth, incubated at 28 °C, and then visualized. (**D**) Growth-kinetics assay after FT2 exposure. Exponential yeast cells exposed to repeated FT cycles were harvested by centrifugation and resuspended into YPD broth. Optical density was measured at 600 nm every 1 h for 12 h. WT, wild-type cells with empty vector; DM, *DaMDHAR*-expressing transgenic cells.

A cDNA containing the open reading frame (ORF) of *MDHAR* from the Antarctic hairgrass *D*. *antarctica* (*DaMDHAR*) was subcloned into the yeast expression vector p426GPD, thereby facilitating the constitutive expression of the *DaMDHAR* gene under the regulation of the yeast *GPD* promoter (Figure 1A). To determine whether the *DaMDHAR* gene is effectively expressed in transgenic yeast, immunoblotting was performed. A single signal intensity corresponding to a region within ~45 kDa) was detected from transformed yeast cells with the p426GPD-*DaMDHAR* construct (DM), whereas there were no intensity in wild-type (WT) yeast cells with vector alone (Figure 1B). To investigate if heterologous *DaMDHAR* expression is related to stress tolerance in yeast cells under FT conditions, a spotting assay was introduced. The spotting assay demonstrated that DM cells had acquired tolerance to FT stress compared to WT cells, dependent on the cycle of FT stress from FT1 to FT6, although stress resistance decreased with progression in the FT cycle. There was a distinct difference in both DM and WT cells under FT stress conditions despite there being no difference under normal conditions (Figure 1C). To confirm these results, growth kinetics was performed. In the growth-kinetics assay, DM cells recovered more rapidly than WT cells for the indicated times when exposed to FT2 stress (Figure 1D). These results suggested that *DaMDHAR* expression in *S*. *cerevisiae* effectively enhances stress tolerance when yeast cells are challenged to FT stress.

### 3.2. Overview of Transcriptome-Based Gene Expression Profiling

**Figure 2:**
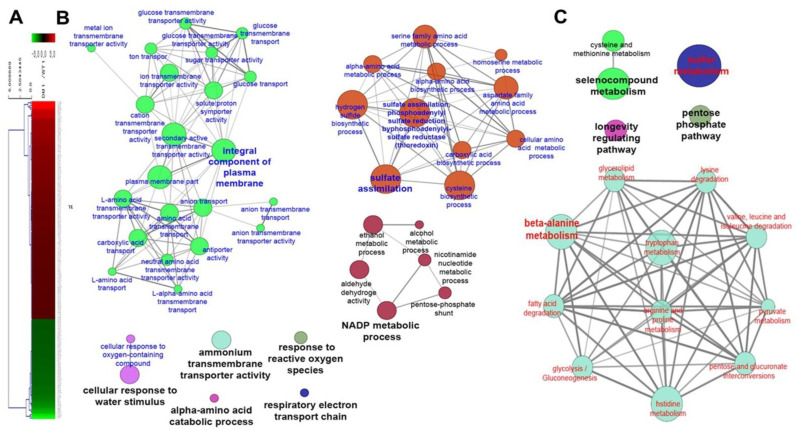
Heat map (**A**) and gene cluster via genetic network analysis based on gene ontology (GO) (**B**) and Kyoto Encyclopedia of Genes and Genomes (KEGG) (**C**) of twofold changed genes in *DaMDHAR*-expressing yeast cells under repeated FT (FT2) stress.

Compared with WT cells, DM cells showed increased survival following acquired tolerance to FT stress. To further elucidate the molecular mechanism underlying the improved tolerance of DM cells, gene expression profiling was assessed by 3′-quant RNA sequencing after exposure to FT2 stress. Of 7126 transcripts identified, 197 genes were showed ≥2-fold changes in DM cells under FT2 stress than in WT cells, whereas 135 genes were downregulated in DM cells under the same condition (Figure 2A). Genes showing 2-fold changes were related to the NADP metabolic process, an integral component of the plasma membrane (PM), sulfate assimilation, cellular response to water stimulus, ammonium transmembrane transporter activity, α-amino acid catabolic process, response to ROS, respiratory electron transporter chain, β-alanine metabolism, selenocompound metabolism, and pentose phosphate pathway (PPP; Figure 2B,C). Furthermore, among the twofold changed genes encoding unknown proteins, compared with WT cells, 98 were upregulated in DM cells under FT2 stress, and 70 genes were downregulated. The genes encoding a hypothetical or uncharacterized protein occupied more than half of the twofold-changed genes in DM cells under FT2 stress (Table S2). The unknown genes identified could improve our understanding of the adaptive mechanisms involved in FT2 stress response and provide information for the improvement of FT2 stress tolerant-yeast strains. Therefore, these results suggested that high *DaMDHAR* expression in transgenic yeast cells enhances acquired tolerance in FT2 stress response by regulating the gene expression of multiple categories, including the NADP metabolic process, integral component of plasma membranes, sulfate assimilation, cellular response to water stimulus, α-amino acid catabolic process, response to ROS, ammonium transmembrane transporter activity, respiratory electron transport chain, β-alanine metabolism, selenocompound metabolism, longevity-regulating metabolism, sulfur metabolism, and PPP.

### 3.3. Identification of Upregulated Genes in DaMDHAR-Expressing Yeast Cells to FT2 Stress

**Figure 3:**
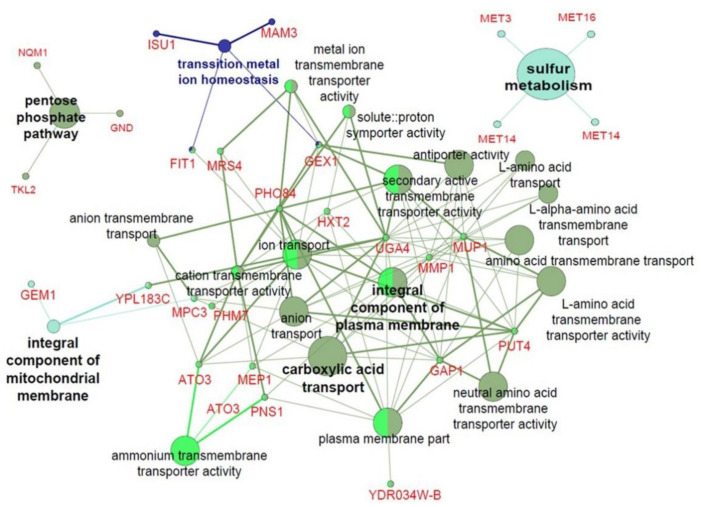
ClueGO-based genetic network analysis of upregulated genes associated with the pentose phosphate pathway, sulfur metabolism, and integral component of mitochondrial component and plasma membrane in *DaMDHAR*-expressing yeast cells under repeated FT (FT2) stress.

**Figure 4:**
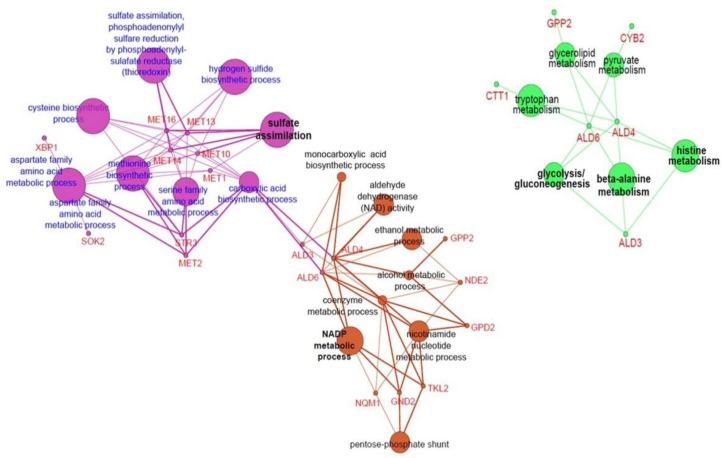
ClueGO-based genetic network analysis of upregulated genes involved in sulfate assimilation, NADP metabolic process, and β-alanine metabolism in *DaMDHAR*-expressing yeast cells under repeated FT (FT2) stress.

Transcriptome analysis using 3′-quant RNA-Seq was used to investigate whether heterologous *DaMDHAR* expression contributes to stress tolerance in FT2 stress by upregulating single gene and gene cluster in transgenic yeast cells. A genetic network analysis was performed using a bioinformatic tool of ClueGO because it performs single cluster analysis, and the comparison of several clusters uses a Cytoscape plug-in that visualizes the non-redundant biological terms for large clusters of genes in a functionally grouped network [50,53]. Among 197 upregulated genes, DM cells induced the expression of genes involved in metal ion homeostasis (*FIT1*, *GEX1*, *ISU1*, and *MAM3*), an integral component of mitochondrial membrane (*GEM1*, *MPC3*, and *YDL183C*), an integral component of PM (*GAP1*, *HXT2*, *MEP1*, *MMP1*, *MUP1*, *PHO84*, *PNS1*, and *UGA4*), carboxylic acid transport (*ATO3*, *GAP1*, *MMP1*, *MPC3*, *MUP1*, *PUT4*, and *UGA4*), sulfate assimilation and reduction, methionine biosynthesis, sulfide biosynthetic process, cysteine biosynthesis, and amino acid metabolic process including aspartate (*MET1*, *MET10*, *MET14*, *MET16*, *MET3, MET12*, *STR3*, *XBP1*, and *SOK2*), NADP metabolic process (*ALD3*, *ALD4*, *ALD6*, *GPP2*, *NDE2*, and *GPD2*), PPP (*GND2*, *NQM1*, and *TKL2*), and β-alanine metabolism (*ALD4*, *ALD6*, and *ALD3*) (Figure 3 and Figure 4; Table 1 and Tables S2–S4). In particular, *ALD4*, *ALD6* and *ALD3* genes of aldehyde dehydrogenase (ALD) were related to amino acids biosynthesis, including histidine, tryptophan, and pyruvate, and glycerolipid metabolism for glycerol synthesis (*GPP2*) as well as glycolysis/gluconeogenesis (Figure 4; Table S4). Interestingly, *CTT1* encoding cytosolic CAT networked to the cluster of β-alanine metabolism via tryptophan metabolic process.

Besides genetic network, the activated genes are as follows: *FRE8* encoding putative ferric-chelate reductase, *GTO3* encoding ω-class GSH transferase, *PUT4* encoding proline permease, *CTT1* encoding cytosolic CAT, *YOL024W* encoding thiol-sulfide oxidoreductase active site, *GEX1* encoding proton–GSH exchanger, *SPG8* encoding protein required for survival against heat shock, *GAP1* encoding general amino acid permease, *ATO3* and *MEP1* encoding ammonium permease, *PDR15* encoding ATP-binding cassette multidrug transporter, and *SIP18* encoding phospholipid-binding hydrophilin (Table 1 and Table S2).

By contrast, *DaMDHAR* overexpression in DM cells upregulated transcription factors (*CIN5* and *MSN4*), putative maltose-responsive transcriptional factor (*YPR196W*), and transcription repressor (*XBP1* and *ROX1*) under FT2 stress (Table 1). With respect to organelles, DM cells also upregulated multiple genes involved in the functionality and stabilization of mitochondria (*ALD4*, *CRC1*, *FMP45*, *GEM1*, *NDE2*, *MRS4*, *SCM4*, *DPI8*, *YDL183C*, *MBR1*, *ISU1*, *MIN3*, *MAM3*, *SHH4*, and *MPC3*), PM (*YDR034W-B*, *BDH2*, *HSP12*, *SIP18*, *YNL195C*, *PHO89*, *PDR15*, and *ATO3*), and cell wall integrity (CWI) and remodeling (*CWP1*, *SPI1*, *PIR3*, *MNN4*, and *FIT1*) (Figure 3; Table 1 and Tables S2–S4). Of the 197 upregulated genes, the highlighted gene was *RCK1* encoding protein kinase involved in oxidative stress (Table 1); further, 98 genes encoding hypothetical or uncharacterized protein were identified in DM cells under FT2 stress (Table 1 and Table S2). The gene expression of *YJL136W—A* hypothetical encoding protein was the lowest, following that of *RCK1* (Table 1). Cumulatively, these results indicated that heterologous *DaMDHAR* expression in transgenic yeast cells facilitates the functions of mitochondria, PM, and cell wall stabilization by enhancing redox, ion, nutrient and water homeostasis, reducing power NAD(P)H—generating system, and sulfate assimilation via the activation of transcriptional factor, protein kinase, and signaling sensor under FT2 stress.

### 3.4. Screening of Downregulated Genes in DaMDHAR-Expressing Yeast Cells to FT2 Stress

**Figure 5:**
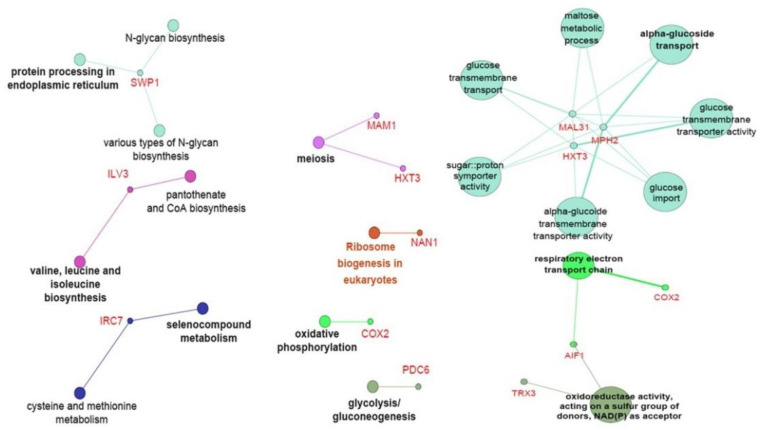
ClueGO-based genetic network of downregulated genes in *DaMDHAR*-expressing yeast cells under repeated FT (FT2) stress.

Compared with *DaMDHAR*-dependent upregulated genes, downregulated genes were identified in FT2 stress. Among the 135 downregulated genes, compared with WT cells, DM cells expressing *DaMDAHR* decreased the expression of genes involved in protein processing in the endoplasmic reticulum (ER; *SWP1*), α-glucoside transport connected with glucose transport, sugar::proton symporter, α-glucoside transporter, and maltose metabolic process (*MAL31*, *MPH2*, and *HXT3*), respiratory electron transport chain (*AIF1* and *COX2*), oxidoreductase activity using NAD(P)^+^ as an acceptor (*TRX3*), pantothenate CoA biosynthesis (*ILV3*), selenocompound metabolism (*IRC7*), meiosis-dependent cell cycle combined with *HXT3* and *MAM1*, ribosome biogenesis (*NAN1*), oxidative phosphorylation (*COX2*), and glycolysis/gluconeogenesis (*PDC6*), under FT2 stress (Figure 5; Table 2, Tables S2, S5, and S6). *COX2* encoding cytochrome c oxidase was connected to the co-cluster of oxidative phosphorylation and respiratory electron chain (Figure 5). In particular, *MAM1* (coding monopolin) and *HXT3* (coding low-affinity glucose transporter) were downregulated in DM cells under FT2 stress, which was connected with meiosis regarding KEGG-based classification (Figure 5 and Table S6). In addition to *MAM1* coding meiosis-specific kinetochore-associated protein, a low mRNA level of *HXT3* provides a growth advantage for young daughters during mitosis or quiescence under FT2 stress, who better prepared for nutritional changes in FT2 stress. By contrast, other *HXT* genes, such as *HXT2* and *HXT4* with high-affinity glucose transporter, were activated or unchanged during the mitosis or quiescence cell cycle under stress conditions, which led to glucose homeostasis. Thus, these results indicate that *DaMDHAR*-expressing DM cells could, at least partially, enhance the mitotic cell cycle with cell division or quiescence arrest, but not the meiosis-dependent cell cycle. With regard to the single gene, DM cells decreased the gene expression of *AIF1* encoding mitochondrial cell death effector, *YNL190W* encoding hydrophilin, *YPK2* encoding putative protein kinase similar to S/T protein kinase Ypk1p, *VPS26* encoding vacuolar protein sorting, *TRX3* encoding mitochondrial thioredoxin, *SSH4* encoding a specific factor required for ubiquitination, *HSP30* encoding heat shock protein (HSP) 30, *COX2* encoding cytochrome c oxidase subunit II, and *FDH2* encoding NAD^+^—dependent formate dehydrogenase (Table 2 and Table S2). *HSP30* expression was the highest low in DM cells (Table 2).

In relation to organelles, DM cells downregulated the expression of genes related to functionality and metabolism of mitochondria (*ILV3*, *TRX3*, *COX2*, *AIF1*, and *OCD2*), PM (*ZEO1*, *MAL31*, *RIM19*, *MPH2*, *HXT3*, *HXT17*, *SMA1*, and *FSP30*), and CWI (*YNL190W* and *NCW1*) (Table 2, Tables S2, S5, and S6). Regarding transcription factors, compared with WT cells, DM cells downregulated putative transcriptional activator (*MSN2*), zinc finger transcriptional repressor (*MIG2*), and *MGA1* encoding protein similar to heat shock transcriptional factor under FT2 stress compared to WT cells (Table 2 and Table S2). By contrast, 70 genes encoding a hypothetical or uncharacterized protein decreased in DM cells under FT2 stress, and the expression of *YJL197-C* was the lowest following that of *HSP30* (Table 2 and Table S2). Thus, these results suggested that *DaMDHAR* expression contributed to FT2-mediated stress response by regulating oxidative phosphorylation, selenocompound metabolism, cell cycle arrest, and ER and ribosomal functions via transcriptional activator and repressor.

### 3.5. Determination of Cell Rescue Systems and NADPH Concentration

**Figure 6:**
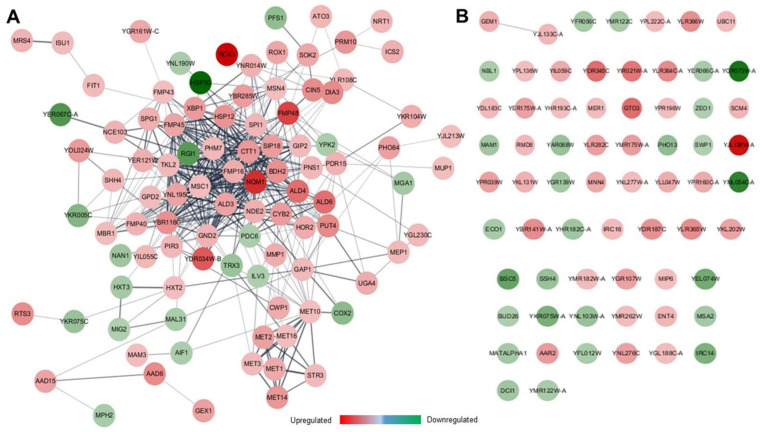
StringGO-based genetic network of multiple genes (**A**) and single gene (**B**) of twofold changed genes in *DaMDHAR*-expressing yeast cells under repeated FT (FT2) stress.

The total network of twofold changed genes, including cell rescue systems, was visualized via StringGO-based genetic interaction. As shown in Figure 6, approximately half of the two-fold-changed genes formed a genetic network; however, the rest remained networked. Stress-responsive genes, including *FMP16* encoding protein involved in responding to stress conditions, *PHM7* encoding vacuolar transport chaperone, *MSC1* encoding protein with ubiquitin ligase activity, *NQM1* encoding transaldolase, *CTT1* encoding cytosolic CAT, and *ALD3* encoding ALD, formed the central core of the genetic network. Future studies of the genetic interaction for the unconnected single genes should be entailed to further elucidate the sophisticated tolerance mechanism to FT2 stress.

**Figure 7:**
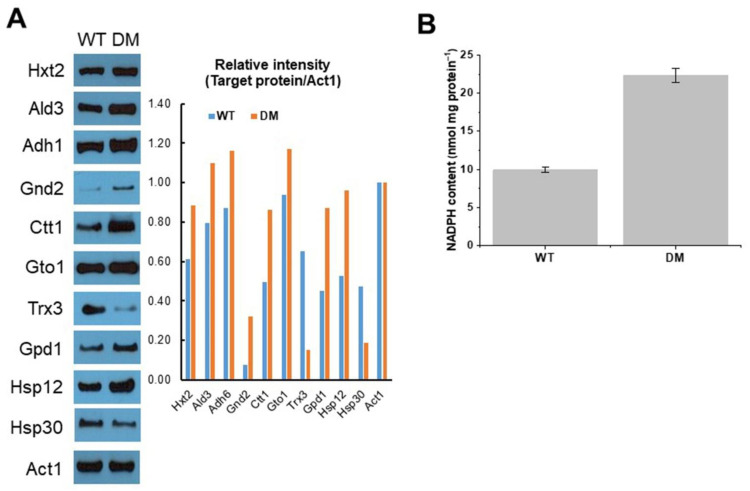
Expression analysis at the protein level of 2-fold changed genes (left panel) and signal intensity (right panel) (**A**) and NADPH assay (**B**). Signal intensity was calculated using Image J. Evaluation of intracellular NADPH was assayed by spectrophotometric quantification. Data were analyzed using Origin 2020. The data are the means and standard deviations of three independent experiments with at least two biological replicates each. A probability analysis to determine statistical significance was performed using the Student’s *t*-test. Compared to the appropriate control strain, *p* < 0.05 was considered significant in this study. Hxt2, hexokinase 2; Ald3, aldehyde dehydrogenase isoform 3; Adh6, alcohol dehydrogenase isoform 6; Gnd2, 6-phosphogluconate dehydrogenase; Ctt1, cytosolic catalase; Gto1, glutathione-S-transferase isoform 1; Trx3, Mt thioredoxin; Gpd1, glyceraldehyde-3-phosphate dehydrogenase; Hsp12, heat shock protein 12; Hsp30, heat shock protein 30; Act1, actin isoform 1 was used as loading control; WT, wild-type cells with empty vector; DM, *DaMDHAR*-expressing transgenic cells.

Thereafter, immunoblotting analysis and NADPH assay were performed to demonstrate the change from transcriptional to translational levels. As shown in Figure 7, compared with WT cells with empty vector alone, protein expression coding hexokinase (Hxt2), ALD, alcohol dehydrogenase (Adh6), 6-phosphogluconate dehydrogenase (Gnd2), CAT (Ctt1), GSH transferase (Gto1), GAPDH (Gpd1), and Hsp12 showed high accumulation in DM cells expressing *DaMDHAR* under FT2 stress, whereas the protein level encoding thioredoxin 3 (Trx3) and Hsp30 in DM cells decreased under FT2 stress (Figure 7A). Protein expression patterns mimicked the changes in mRNA expression of each gene, despite the imperfect relationship between mRNA and protein level observed. The moderate differences observed between mRNA and protein levels were likely due to post-transcriptional modification. The difference between mRNA and protein levels was high for Hsp30, suggesting that the degraded Hsp90 protein was capable of binding to Hsp30.

Thereafter, we estimated the intracellular NADPH levels in WT and DM cells under FT2 stress using spectrophotometric quantification. The NADPH concentration of DM cells was 2.25-fold higher than that of WT cells under the same stress (Figure 7B). High NADPH levels resulted from gene activation associated with the PPP. This increase in NADPH in DM cells likely reflected the overall levels, including changes of cytosol and mitochondrial compartment. Therefore, these results suggested that gene expression provides, at least partially, a direct relationship between mRNA transcripts and protein levels.

### 3.6. Cellular Response to Abiotic Stresses and Importance of D-Erythroascorbate as a Substrate

**Figure 8:**
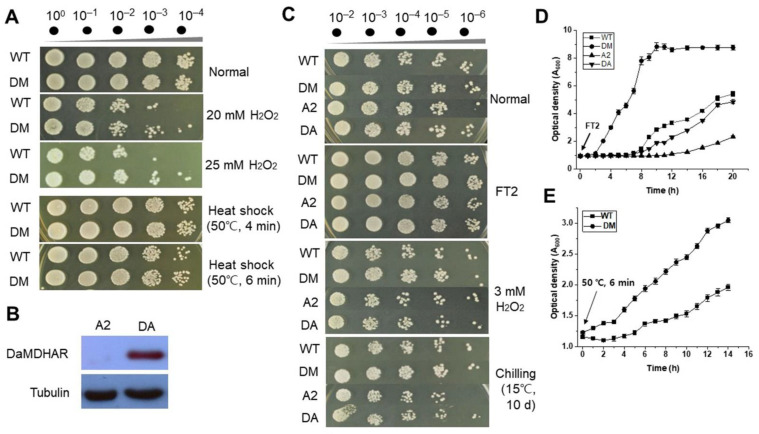
Cellular response in *DaMDHAR*-expressing yeast cells to oxidative stress and heat shock. (**A**) Exponential yeast cells (A_600_ ≈ 1.5) were exposed to 20 and 25 mM H_2_O_2_ for 1 h at 28 °C with shaking (160 rpm) and diluted to 10^−4^ with YPD broth. For heat shock, the same-phase yeast cells were challenged for 4 and 6 min at 50 °C. Five microliters of the diluted solution were spotted onto the YPD agar broth, incubated at 28 °C, and then visualized. (**B)** Construct in *DaMDHAR*-expressing yeast cells using D-erythroascorbate (eAsA) instead of ascorbate as a substrate. The deleted strain (*ara2*Δ) of the *ARA2* gene encoding NAD-dependent arabinose dehydrogenase was employed. After establishing *DaMDHAR*-expressing *ara2*Δ (DA) cells and *ara2*Δ (A2) cells with empty vector alone via transformation, protein expression in both cells was confirmed by western blot analysis. (**C**) Cellular response assay to abiotic stress was performed using the spotting assay. Exponential yeast cells (A_600_ ≈ 1.5) grown in YPD broth were serially diluted to 10^−6^ with YPD broth. Five microliters of each diluted solution were spotted onto the YPD agar medium in the absence and presence of 3 mM H_2_O_2_ and incubated for 3 days at 28 °C or 10 days at 15 °C. For FT, exponential cells were exposed to repeated FT cycles (FT2), diluted with YPD broth, spotted onto the YPD agar medium, and incubated for 3 days at 28 °C. (**D**) Growth kinetics after FT2 stress. Log-phase yeast cells exposed to FT2 stress were resuspended into a fresh YPD broth medium and incubated at 28 °C for 20 h with shaking (160 rpm). Optical density was measured every 2 h at 600 nm. (**E**) Cellular response to heat shock in *DaMDHAR*-expressing yeast cells. Log-phase yeast cells exposed to heat shock at 50 °C for 6 min were harvested by centrifugation and resuspended into a fresh YPD broth medium. Optical density was measured at 600 nm in 1 h-interval for 14 h. WT, wild-type cells with empty vector; DM, *DaMDHAR*-expressing transgenic cells. WT, wild-type cells with empty vector; DM, *DaMDHAR*-expressing transgenic cells; A2, *ara2*Δ cells with empty vector alone; DA, *DaMDHAR*-expressing *ara2*Δ cells.

It was examined whether *DaMDHAR* expression in transgenic yeast affects the cellular response to oxidative stress and high-temperature. As shown in Figure 8, the survival of *DaMDHAR*-expressing DM cells was better than that of WT cells when exposed to 20 and 25 mM hydrogen peroxide (H_2_O_2_) for 1 h at 28 °C, or challenged for 4 and 6 min at 50 °C. Further, AsA is the most common antioxidant molecule in the cell; thus, it is a good target for investigating the acquired ability of *DaMDHAR*-expressing cells to detoxify ROS produced by abiotic stress [19,21]. Yeasts do not typically synthesize AsA; instead, they synthesize a five-carbon analog, D-erythroascorbic acid (eAsA), from D-arabinose by D-arabinose dehydrogenase (ARA2) and D-arabino-c-lactone oxidase activity. This eAsA shares structural and physiochemical properties with AsA, including roles in regulating antioxidant activity, hyphal growth, and stress tolerance. Unlike, AsA, the function of eAsA in cellular stress response is mostly unknown. Therefore, we used a yeast strain in which the eAsA biosynthesis-related gene ARA2 was deleted (*ara2*∆) to demonstrate whether eAsA deficiency in *ara2*∆ affected the cellular response to FT stress. A functional examination of *ara2*∆-mediated eAsA was confirmed by evaluating empty vector (p426GPD)- and DaMDHAR::p426GPD-transformed *ara2*∆ cells (named as A2 and DA, respectively, Table S1) via immunoblotting analysis (Figure 8B). The stress-response assay was performed by spotting assay using WT, DM, A2, and DA. Yeast cells that reached the early exponential phase were serially diluted with YPD broth medium. For H_2_O_2_ and low-temperature assay, 5 µL each of the diluted solution was spotted onto the YPD agar medium in the absence and presence of 3 mM H_2_O_2_. FT2 stress was performed as described in Materials and Methods. Among the four transgenic yeast cells, stress tolerance was observed in the order of DM, WT, DA, and A2 under FT2, H_2_O_2_, heat, and chilling stress. There was no difference in stress response between A2 and DA cells (Figure 8C–E). Therefore, these results indicated *DaMDHAR* expression facilitates enhanced tolerance to abiotic stress, including H_2_O_2_ and low-temperature, implying that eAsA plays a critical role in the *DaMDHAR*-mediated abiotic stress response.

## 4. Discussion

### 4.1. Phenotype-Based Stress Resistance of DaMDHAR-Expressing Yeast Cells to FT

Freeze-tolerant aerobic organisms, including yeasts, must survive ice formation as well as ROS generation during oxygen resupply in thawing [54]. Variation in extreme temperature places yeasts at risk of being challenged to multiple FT cycles. However, few studies have reported a relationship between tolerance and oxidative stress in yeasts exposed to freezing conditions [54,55]. Therefore, there is a need to investigate the molecular mechanism that underlies FT damage and response by cells to prevent it [55]. To explore this, we created transgenic *S*. *cerevisiae* expressing the *DaMDHAR* gene from the Antarctic hairgrass plant. We used this artificial system to investigate the functionality and regulatory properties of *DaMDHAR* in FT stress response. The stress response in *DaMDHAR*-expressing transgenic yeast cells following treatment with six-repeated cycles of freezing at −70 °C and thawing at 25 °C, was assessed. DM cells expressing *DaMDHAR* were tolerant to FT stress than WT cells with empty vector alone, although cell viability decreased in an FT-dependent manner (Figure 1C,D). Similarly, *MDHAR* overexpression increased acquired tolerance to abiotic stress, including salt, chilling, and oxidative stress, in transgenic microalgae [56], yeast [41], *Arabidopsis* [57], tomato [58], and tobacco [59].

By contrast, DM cells improved enhanced resistance to abiotic stress, including H_2_O_2_ and high-temperature (Figure 8A and Figure S1). In particular, no differences were observed between DA cells expressing *DaMDHAR* and A2 cells with empty vector alone under FT2 stress (Figure 8C). As mentioned above, *S*. *cerevisiae* synthesizes an isomeric D-form of eAsA as a five-carbon homolog of AsA. eAsA has a crucial role in antioxidant activity and cyanide-resistant respiration [60,61,62]. In the antioxidant process, eAsA is oxidized to *tert*-butylhydroperoxide radical, but not H_2_O_2_, which is reduced by NADH-cytochrome b5 reductase (Mcr1p) and Gtop with DHAR activity [63] as well as by DaMDHAR, considering that MDHAR is capable of reducing a wide range of radicals, including AsA, MDHA, quinone, and phenoxyl radicals [62,64,65]. Thus, these results suggested that *DaMDHAR* improves acquired tolerance to abiotic stresses, particularly FT stress, using eAsA instead of AsA as a substrate.

### 4.2. Regulation of Transcription Factors in DaMDHAR-Expressing Yeast Cells under FT2 Conditions

The mechanism of yeast stress tolerance involve various aspects of growth control, sensing, and signal transduction, as well as the regulation of transcription, translation, and posttranslational modifications [43]. Several results have explained the damage generated by FT based on an analysis of the physiochemical changes that occur in cells [55]. However, understanding the FT-mediated tolerance mechanism is complicated and not well established. To demonstrate the *DaMDHAR*-mediated stress tolerance mechanism to repeated FT cycle (FT2), a transcriptome-based gene expression profiling and the genetic network were performed using the ClueGO tool. DM cells expressing *DaMDHAR* upregulated transcription factors and their components (*CIN5* and *MSN4*), transcriptional repressor (*XBP1*), and putative maltose-responsive transcription factor (*YPR196W*), but downregulated *MGA1* encoding protein similar to heat shock transcription factor, *MSN2* encoding putative transcription factor in stress response, and *MIG2* encoding zinc-finger transcriptional repressor (Table 1 and Table S2).

In particular, DM cells activated the transcription factor *MSN4* under FT2 stress, but not *MSN2* (Table 1). Msn2/4p transcription factors are induced in response to various environmental changes, including H_2_O_2_ [66]. *MSN2*, but not *MSN4*, overexpression conferred improved tolerance to oxidants, high concentration of ethanol, acid and alkaline stress, and salinity [67,68,69,70,71] and the deletion and downregulation of *MSN2* generate a dramatic decrease in Ctt1p expression [72]. In contrast to the previous reports, these results showed that upregulated *MSN4* in DM cells increases *CTT1* expression under FT2 stress but not downregulated *MSN2* (Table 1). Cumulatively, these results suggested that the *DaMDHAR*-dependent resistance mechanism to FT stress is mediated by the activation of *MSN4* and *CIN5* and repression of *XBP1*.

### 4.3. Regulation of the Protein Kinase-Mediated Signaling Pathway in FT2 Stress

*DaMDHAR*-expressing DM cells upregulated the gene expression of *RCK1* encoding protein kinase involved in oxidative stress and *MET14* encoding adenylyl sulfate kinase for sulfate assimilation and methionine metabolism under FT2 stress; however, they downregulated *YPK2* encoding protein kinase similar to S/T protein kinase Ypk1p (Table 1 and Table S2). As shown in Figure 5, *RCK1* activation inhibits meiosis in yeast [73] and increases enhanced tolerance to oxidative stress and acetic acid [74,75]. Accordingly, the activated expression of the *RCK1*-dependent MAPK (*HOG*) gene conferred tolerance to salinity and FT stress [76]. In addition to *RCK1*, meiosis repression under FT2 stress was involved in a downregulation of the *MAM1* gene encoding monopolin, a meiosis-specific kinetochore-associated protein (Table S2). The relationship between phenotypes and genes regulating the growth phase has not been extensively investigated.

*S*. *cerevisiae* generates sulfite as an intermediate product during the assimilatory reduction of sulfate to sulfide [77]. Three genes, *MET3* encoding ATP sulfurylase, *MET 16* encoding thioredoxin-dependent phosphoadenylyl-sulfate reductase, and *MET14* are essential for the sulfur assimilation process (Figure 3). *MET14* overexpression increases sulfite formation by reducing cellular sulfate concentration [77], and then the reduced sulfite can contribute to cellular stress response by combining with *MET10* encoding sulfite reductase overexpressed under FT2 stress [77]. In addition, the TORC-Ypk1/Ypk2 kinase cascade in yeast plays a critical role in PM stress response by remodeling the actin cytoskeleton, although the kinase cascade-dependent molecular mechanism is not well-known [78]. These results showed that stress tolerance to FT2 is independent of the Ypk2 kinase pathway as *YPK2* is downregulated in a *DaMDHAR*-dependent cellular response to FT2 stress.

### 4.4. Enhancement of Cell Rescue Systems in FT Stress

Cell rescue systems, including redox homeostasis, ion homeostasis, and proteostasis, play critical roles in stress response to abiotic stress, including FT [79]. In relation to enzymatic antioxidant systems, DM cells upregulated *CTT1* encoding cytosolic CAT, *GTO3* encoding ω-class GSH transferase, and *YOL024W* encoding hypothetical protein predicting to have thiol-disulfide oxidoreductase active site under FT2 stress, but downregulated *TRX3* of mitochondrial thioredoxin and *AIF1* encoding mitochondrial cell death effector (Figure 7A; Table 1 and Table S2). The involvement of oxidative stress response in FT has been investigated using yeast mutants defective in different antioxidant functions (mainly superoxide dismutase) but not using high-throughput analysis [43]. Among the activated genes, *CTT1* encoding cytosolic CAT protects cells from oxidative damage by converting toxic H_2_O_2_ to H_2_O [80]. Cytosolic GTO3 is one of the three ω-class GSH transferases with the enzymatic activity of this transferase (glutaredoxin), DHAR, and dimethylarsinic acid reductase and is functionally related to sulfur metabolism [81,82]. In addition, DM cells upregulated the *AAD6* gene under FT2 stress (Table S2). The protein-encoding aryl-ADH, including *AAD6*, may play a critical ancestral role in neutralizing aromatic aldehyde in yeast [83]. By contrast, *TRX3* was downregulated in DM cells under FT stress (Figure 5 and Figure 7A; Table 1 and Table S2). Mitochondrial Trx3 functions as an antioxidant defense system, and its oxidation promotes cell death via apoptosis in response to oxidative stress [84]. Altogether, these results showed that aerobic FT2-responsive injury in *DaMDHAR*-expressing transgenic yeast cells is caused by oxidative stress, and this process was mainly initiated in the cytoplasm via an oxidative burst of H_2_O_2_ or free radicals produced from oxygen and electron released from the mitochondrial electrons transport chain, thereby supporting that redox homeostasis is achieved by CAT and thiol-containing antioxidant enzymes.

With respect to HSPs, DM cells expressing *DaMDHAR* showed a high accumulation of *HSP12* transcript but reduced *HSP30* expression (Figure 7A; Table 1 and Table S2). Hsp12, as a major small sHSP, contributes to stress tolerance to freezing conditions (−20 °C) [85]. Although Hsp30, a PM-localized HSP, is induced by various abiotic stressors, its expression was downregulated in DM cells under FT2 stress. Hsp30 downregulation activates Pma1p with PM H^+^-pump activity under high-temperature stress [86,87]. Accordingly, *SWP1* and *NAN1* involved in protein folding/refolding were downregulated in FT2-treated DM cells compared with WT cells (Figure 5). Molecular chaperones enhanced the refolding of denatured proteins under FT stress, acting as a general stress resistance mechanism [43]. These results indicated that an enhanced understanding of the cellular expression of these sHSPs could shed light on the biological role or physiological characteristics of FT stress.

### 4.5. DaMDHAR-Dependent Metal Ion Homeostasis in FT2 Stress

Under FT2 stress, compared with WT cells, DM cells increased the expression of genes involved in metal ion homeostasis (*GEX1* and *ISU1*) via ion transport (*MRS4*, *PHO89*, and *YDL183C*). DM cells upregulated *FRE3* encoding protein similar to iron/copper reductase (Figure 3; Table 1 and Table S2). Reports regarding a relationship between FT stress and ion homeostasis are scarce. *GEX1* induced by iron depletion functions in the proton–GSH antiporter system [88]. *ISU1* overexpression augments ethanol tolerance by decreasing ROS and free-iron accumulation during ethanol fermentation [89]. *MRS4* expression encoding the mitochondrial carrier family regulates excess iron accumulation in the mitochondria and is directly involved in mitochondrial iron uptake under iron-limiting conditions [90]. The Na^+^/phosphate transporter PHO89 is functional to sustain growth under high pH stress [91]. Defects of *YDL183C* overexpressed in DM cells under FT2 stress increase K^+^ content, swelling, and autophagic decay of the mitochondria, whereas its overexpression rescues cell growth and K^+^/H^+^ exchanger activity [92]. *FRE3* gene induced in FT2-treated DM cells is expressed either in cells exposed to limited conditions of iron uptake or in high-affinity iron uptake-deficient cells, likely involving copper and iron homeostasis [93,94]. Hence, these results indicate that high metal ion concentration, particularly iron, is trafficked into the mitochondria using iron-sulfur cluster assembly and other chelating systems, including mitochondrial carrier and metalloreductase, thereby leading to metal ion homeostasis in organelles, particularly mitochondria.

### 4.6. Regulation of β-Alanine Metabolism in DaMDHAR-Expressing Yeast Cells to FT2 Stress

Gene expression related to β-alanine metabolism (*GPP2*, *ALD3*, *ALD4*, and *ALD6*) and water-responsive channel (*SIP18*) was upregulated in DM cells under FT2 stress compared with WT cells (Figure 4; Table 1). *GPP2* and *SIP18* encode DL-glycerol-3-phosphate phosphatase in glycerol biosynthesis and phospholipid-binding hydrophilin, respectively, which are essential to overcome the dehydration-rehydration process. Water/glycerol channels are known as crucial in life processes, such as FT tolerance, and osmoregulation as well as phenomena associated with the cell surface [95]. In addition, upregulation of *ALD2*, *ALD4*, and *ALD6* encoding ALD plays a critical role in protecting against various environmental stressors, such as dehydration, because they exhibit antioxidant activity, self-protection, cell differentiation, or cell population expansion [96,97]. Therefore, the activation of gene expression involved in β-alanine increases stress tolerance by enhancing the mitochondrial redox state via the prevention of water loss and neutralization of toxic aldehyde and H_2_O_2_ under FT2 conditions.

### 4.7. Activation of PPP in FT2 Stress

*DaMDHAR*-expression in transgenic yeast cells activated the expression of genes involved in the NADPH-producing metabolic process (*GND2*, *NQM1*, and *TKL2*) by PPP under FT2 stress compared with WT cells (Figure 4; Table 1 and Tables S2–S4). In particular, 6-phosphogluconate dehydrogenase encoded by *GND2* plays a key role in catalyzing an NADPH regenerating reaction in the PPP. Accordingly, a high expression of these genes in DM cells leads to an increased NADPH pool (Figure 7B). Increased cellular NADPH pool is important for redox biology; NADPH plays a crucial role in the functions of antioxidant enzymes, including peroxiredoxins, thioredoxin peroxidase, GSH peroxidases, and CAT [98,99].

### 4.8. DaMDHAR-Mediated Mitochondrial Stabilization at the Transcriptional Level against FT

Mitochondrial functionality is a crucial factor in cell survival associated with FT stress tolerance in yeast and is elaborately connected to the mitochondrial metabolism process [100]. In addition to *TRX3*, *ISU1*, *MRX4*, *ALD6*, and *AIF1*, DM cells upregulated multiple genes involved in mitochondrial functionality and mitochondrial metabolism under FT2 stress compared with WT cells, but they downregulated *ILV3* and *OCD2* expression under the same conditions (Figure 3 and Figure 5; Table 1, Table 2, and Table S2). Upregulated *CRC1* encodes a carnitine carrier protein that transports carnitine, acetylcarnitine, and poly-chain acylcarnitines [101]. *FMP45* and *GEM1* encode an integral membrane protein to maintain sphingolipid and phospholipid content, respectively, in yeast [102,103,104]. Mitochondrial NADH dehydrogenase encoded by *NDE2* and *NDE1* oxidizes NADH at the cytosolic side of the inner membrane, and oxidized NAD^+^ is recycled by both Nde1/2 and glycerol synthesis shuttle composed of Gpd1/2 and Gut2 in the presence of respiratory chain [105,106]. Furthermore, *GPD2* involved in glycerol biosynthesis shuttle was upregulated in DM cells under FT2 stress, encoding NAD-dependent glycerol-3-phosphate dehydrogenase (GAPDH) (Figure 7A; Table S2). Accordingly, freezing stress increases the concentration of the cryoprotectant glycerol [54]. Upregulated *CYB2* encoding cytochrome b2 (L-lactate cytochrome-c oxidoreductase) is a component of the mitochondrial intermembrane space, which controls mitochondrial biogenesis [107]. Thus, these results suggest that a connection between Mt function and FT stress tolerance.

### 4.9. DaMDHAR-Dependent PM Stabilization at the mRNA Level against FT

PM is frequently challenged to abiotic stresses. To minimize stress-mediated PM damage, aerobic cells have a broad range of defense systems. Considering these facts, *DaMDHAR*-dependent cell rescue systems on PM were explored under FT2 stress. In relation to PM, *DaMDHAR*-expressing DM cells upregulated a wide range of genes under FT2 stress compared with WT cells, but they downregulated *ZEO1*, *MAL31*, *RIM9*, *MPH2*, *HXT3*, *SMA1*, *HXT17*, and *HSP30* under the same conditions (Figure 3, Figure 4 and Figure 5 and Figure 7A; Table 1 and Table 2 and Tables S2–S6). In yeast, several sensing systems localized to PM regulate the expression and activity of a protein involved in nutrient uptake and metabolism [108]. For instance, gene activation encoding various nutrient permeases, including the ammonium permease Mep1, methionine permease Mup1, and general amino acid permease Gap1, regulates nutrient homeostasis and cyclic AMP-mediated fast energy recycling to overcome environmental stress such as FT [109].

Overexpressed *PUT4* encoding specific proline permease facilitates stress resistance to heat shock and osmolarity via proline uptake [110]. *UGA4* encoding γ-aminobutyric acid and δ-aminolevulinic acid permease are regulated by extracellular amino acids condition [108]. Upregulated *ATO3* encoding a putative PM ammonium transporter is associated with eliminating the excess ammonia produced during amino acid metabolism via the outward ammonia release [111]. By contrast, downregulated *RIM9* is considered an auxiliary component of the pH sensor machinery upon external alkalization [112]. The downregulation or deletion of *HSP30* abolished the pH buffering activity instead of shifting cytosolic pH [87]. High change expression of these genes may rapidly regulate the cellular nutrients and pH homeostasis between the PM and other organs *SIP18* with PM phospholipid-binding hydrophilin activity wad upregulated in DM cells under FT2 stress, whereas *YNL190W* with cell wall hydrophilin activity was downregulated under the same condition (Table 1 and Table 2). *SIP18*-dependent genetic improvement prevented cell death during desiccation and freezing stress [113,114,115]. In addition, upregulated *HSP12* belonging to the hydrophilin family can reinforce stress tolerance by preventing severe water loss under desiccation stress [116]. By contrast, *YNL190W* downregulated in DM cells is induced in response to organic solvents [117,118]. The expression combination of *SIP18* and *HSP12* genes could maximize rehydration from environmental stress causing water loss such as FT2 stress. By contrast, highly accumulated *PDR15* and *YKR104W* expression encoding a multidrug-resistant protein enhance stress resistance to intracellular xenobiotics produced during FT2 stress, and their inactivation increase xenobiotic sensitivity [119,120]. *DaMDHAR* expression confers stress tolerance by preventing dehydration and detoxifying toxic xenobiotics triggered during FT2 stress. Thus, these results indicate that the maintenance of PM integrity after physical damage is critical for FT stress response; retaining proper membrane function and flexibility increases cell survival by allowing more efficient dehydration.

### 4.10. Changed Genes Associated with the Cell Wall in DaMDHAR-Expressing Yeast Cells to FT2 Stress

*DaMDHAR*-expressing DM cells were induced to respond to FT2 stress to upregulate genes encoding the cell wall-related proteins Mnn4p, Cwp1p, Pir3p, and Fit1p compared to WT cells, but they were decreased in the same stress response to downregulate genes encoding Ynl190wp and Ncw1p (Table 2 and Table S2). Upregulated *MNN4* and *CWP1* may be directly related to the mannosyl phosphate transfer reaction conferring a net negative charge in the cell wall mannoprotein in yeast, thereby regulating the leakage of proteins from the periplasmic space and import of macromolecules [121,122]. Additionally, high *FIT1* expression encodes a mannoprotein that is incorporated into the cell wall, and its deletion results in the abolished uptake of iron bound to siderophores [123]. As expected, Fit1p may play a role in regulating the concentration of iron associated with the cell wall and PM.

**Figure 9:**
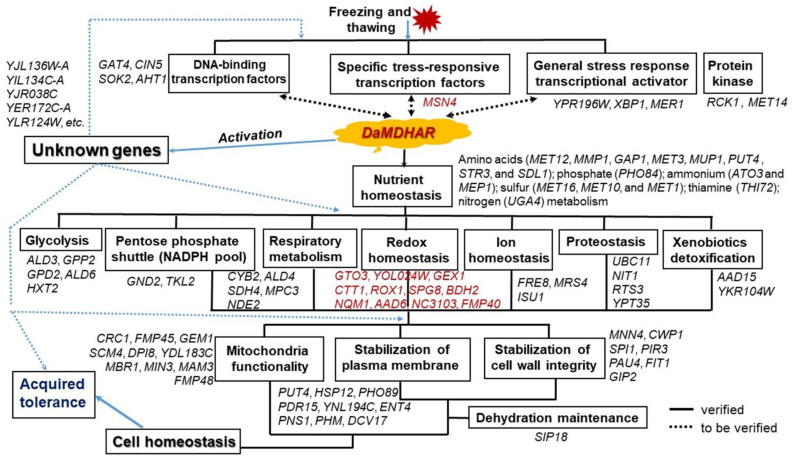
Summary diagram of the adaptive molecular mechanism involved in FT2 stress response in *DaMDHAR*-expressing yeast cells.

Upon stress exposure, the CWI pathway induces the expression of specific cell wall-remodeling genes to build the adaptive strength of the cell wall, including *PIR3* encoding O-glycosylated cell wall protein, as shown in the results (Table S2). The adaptive change in the cell wall after CWI-responsive gene expression is critical for protecting yeast cells against FT2-mediated cell wall stress [36]. By contrast, downregulated *YNL190W* is a hydrophilin essential in the dehydration-rehydration process for CWI [124]. At this point, *DaMDHAR*-expressing yeast cells appear to prefer *MNN4* and *CWP1* expression instead of *YNL190W* during the dehydration-rehydration process after repeated FT conditions. Taken together, among the components of the CWI process, cell wall mannoproteins (*MNN4*, *CWP1*, and *FIT1*) and cell wall remodeling factor (*PIR3*) appeared to be particularly involved in the cell wall stress response to FT2 challenge, supporting that *DaMDHAR*-mediated stress tolerance to FT2 conditions is achieved by enhancing cell wall stabilization via the effective rapid expression program of the known and unknown genes. A previous study reported that budding yeast FT tolerance is influenced by growth phase, mitochondrial function, PM and CWI stability, metabolites (trehalose and α-ketoglutarate), protein (aquaporin and N-acetyltransferase), multi-stress tolerance genes, the target of rapamycin, Ras/cyclic adenosine monophosphate, CWI signaling pathways, and the proteasomal catabolic mechanism [43]. However, our results significantly differed from previously published data, with the exception of the organelle stabilization effects. Hence, our data provide new insight into the molecular mechanism of FT stress resistance in yeast (Figure 9).

### 4.11. Activation of Genes Encoding a Hypothetical Protein as a Determinant of FT Tolerance

The functional characterization of hypothetical, uncharacterized, and dubious genes with a full-length open reading frame is one of the major works of the postgenomic era [83]. Although the first *S*. *cerevisiae* genome sequence was deciphered over 25 years ago, approximately 22% of its estimated 6603 ORFs remain to be verified [83]. This study identified the changed expression of 121 genes encoding a hypothetical protein in *DaMDHAR*-expressing DM cells under FT2 stress. Among twofold changed genes, approximately 65 genes were upregulated in DM cells, whereas 39 genes were downregulated in DM cells (Table 1, Table 2, and Table S2). Changed genes accounted for ≥40%. Genes, including *YJL136W-A*, *YIL134C-A*, and *YJR038C*, were upregulated ≥10 times in DM cells under FT2 stress, whereas genes, including *YJL197C-A*, *YHR069C-A*, and *YOR073W-A*, were downregulated ≥10-times under the same condition. The hypothetical genes suggest that these proteins may have, at least partially, played a crucial ancestral role in responding to FT stress in transgenic yeast. Furthermore, these findings provide new insights on the molecular mechanisms of FT tolerance via which hypothetical genes undergo pseudogenization.

## 5. Conclusions

The mechanisms behind FT stress response have not been fully elucidated in yeast, particularly those related to the ways in which different signals are integrated to regulate tolerance responses. We investigated the relationship between response and tolerance mechanism in transgenic yeast expressing *DaMDHAR*. *DaMDHAR*-dependent tolerance was mediated by the rapid, effective gene expression program related to the detoxification of ROS, prevention of water loss, import of amino acids, sugar, phosphate, and iron, the export of toxic ammonia, proton, and xenobiotics, activation of PPP and methionine metabolism, sulfur assimilation, and glycogen synthesis during FT2 stress. As a result, *DaMDHAR*-transgenic yeast cells could maintain a broad range of homeostasis of redox state, ion, phosphate, amino acids, water and sugar, as well as an NADPH pool. In addition, the state of desiccation and oxidative stress is linked with the reversible delay of metabolism due to the FT of cells. The functionality and metabolism of mitochondria and stabilization via the altered structure or composition of PM and cell wall are associated with parameters strongly affecting yeast cell stress resistance during FT exposure and damage to organelles, including mitochondria, PM, and cell wall during FT2 stress can be a key component causing stress sensitivity (Figure 9). In the future, the functionality of unknown genes on cellular stress response needs to be elucidated as various genes encoding the hypothetical protein occupy more than 40% of the identified genes.

## Figures and Tables

**Table 1:** Upregulated genes in *DaMDHAR*-expressing yeast cells to FT stress.

Gene Symbol	Gene	Fold Ratio (DM/WT)	Description
YGL158W	*RCK1*	16.716	Putative serine/threonine-protein kinase RCK1
YJL136W-A		13.785	Hypothetical protein
YIL134C-A		11.969	Hypothetical protein
YJR038C		10.056	Dubious open reading frame
YDR034W-B		6.267	Predicted tail-anchored plasma membrane protein
YLR047C	*FRE8*	5.285	Protein with sequence similarity to iron/copper reductases
YMR251W	*GTO3*	5.008	Omega class glutathione transferase
YOR374W	*ALD4*	4.982	Mitochondrial aldehyde dehydrogenase
YPL061W	*ALD6*	4.828	Cytosolic aldehyde dehydrogenase
YOR100C	*CRC1*	4.597	Mitochondrial inner membrane carnitine transporter
YOR348C	*PUT4*	3.630	Proline permease
YOR028C	*CIN5*	3.522	Basic leucine zipper transcription factor of the yAP-1 family
YFL014W	*HSP12*	3.227	Plasma membrane heat shock 12 protein
YGR088W	*CTT1*	3.076	Cytosolic catalase T
YOL024W		3.018	Predicted to have thiol-disulfide oxidoreductase active site
YMR175W	*SIP18*	2.951	Phospholipid-binding hydrophilin
YCL073C	*GEX1*	2.855	Proton:glutathione antiporter
YMR169C	*ALD3*	2.758	Cytoplasmic aldehyde dehydrogenase
YDL222C	*FMP45*	2.670	Integral membrane protein localized to mitochondria
YBR296C	*PHO89*	2.609	Plasma membrane Na^+^/Pi cotransporter
YKL096W	*CWP1*	2.496	Cell wall mannoprotein
YAL048C	*GEM1*	2.403	Outer mitochondrial membrane GTPase
YDL085W	*NDE2*	2.403	Mitochondrial external NADH dehydrogenase
YDR406W	*PDR15*	2.343	Plasma membrane ATP-binding cassette (ABC) transporter
YKR052C	*MRS4*	2.330	Iron transporter of the mitochondrial carrier family
YER150W	*SPI1*	2.237	GPI-anchored cell wall protein
YGR049W	*SCM4*	2.235	Mitochondrial outer membrane hypothetical protein
YDR384C	*ATO3*	2.207	Plasma membrane protein, putative ammonium transporter
YKL163W	*PIR3*	2.194	O-glycosylated covalently bound cell wall protein
YJL133C-A	*DPI8*	2.183	Delta-Psi-dependent mitochondrial import protein of 8 kDa
YDL183C		2.141	Protein that may form an active mitochondrial KHE system
YDR534C	*FIT1*	2.115	Mannoprotein that is incorporated into the cell wall
YKL093W	*MBR1*	2.103	Protein involved in mitochondrial functions and stress response
YPL135W	*ISU1*	2.096	Conserved protein of the mitochondrial matrix
YMR182W-A	*MIN3*	2.089	Mitochondrial MINi protein of 3 kDa
YOL060C	*MAM3*	2.074	Protein required for normal mitochondrial morphology
YKL062W	*MSN4*	2.061	Stress-responsive transcriptional activator
YGR243W	*MPC3*	2.050	Mitochondrial pyruvate carrier (MPC)
YPR196W		2.018	Putative maltose-responsive transcription factor

WT, wild-type cells with empty vector alone; DM, *DaMDHAR*-expressing transgenic cells.

**Table 2:** Downregulated genes in *DaMDHAR*-expressing yeast cells to FT stress.

Gene Symbol	Gene	Fold Ratio (DM/WT)	Description
YGL209W	*MIG2*	0.498	Zinc finger transcriptional repressor
YNR074C	*AIF1*	0.485	Mitochondrial cell death effector
YMR104C	*YPK2*	0.484	Protein kinase similar to S/T protein kinase Ypk1p
YNL190W		0.480	Hydrophilin essential in the desiccation–rehydration process
YFR055W	*IRC7*	0.462	β-lyase involved in the production of thiols
YCR040W		0.455	Transcriptional co-activator that regulates mating-specific genes
YGR249W	*MGA1*	0.454	Protein similar to heat shock transcription factor
YKR077W	*MSA2*	0.453	Putative transcriptional activator
YCR083W	*TRX3*	0.418	Mitochondrial thioredoxin
Q0250	*COX2*	0.371	Subunit II of cytochrome c oxidase
YOR073W-A		0.135	Dubious open reading frame
YHR069C-A		0.115	Dubious open reading frame
YJL197C-A		0.115	Dubious open reading frame
YCR021C	*HSP30*	0.110	Negative regulator of the H(+)-ATPase Pma1p

WT, wild-type cells with empty vector alone; DM, *DaMDHAR*-expressing transgenic cells.

## Supplementary Materials

The following are available online at https://www.mdpi.com/2073-4425/12/2/219/s1, Table S1: Genotype of *S*. *cerevisiae* strains used in this study, Table S2: Twofold changed genes in *DaMDHAR*-expressing yeast cells to FT stress, Table S3: GO-based classification of upregulated genes in *DaMDHAR*-expressing yeast cells; Table S4: Kyoto Encyclopedia of Genes and Genomes (KEGG)-based classification of upregulated genes in *DaMDHAR*-expressing yeast cells, Table S5: GO-based classification of downregulated genes in *DaMDHAR*-expressing yeast cells, Table S6: KEGG-based classification of downregulated genes in *DaMDHAR*-expressing yeast cells.
