# Supplementary material for: Screening and Genetic Network Analysis of Genes Involved in Freezing and Thawing Resistance in DaMDHAR—Expressing Saccharomyces cerevisiae Using Gene Expression Profiling
Source: Genes (Basel). 2021 Feb 3;12(2):219. doi: 10.3390/genes12020219 (PMC7913288; doi:10.3390/genes12020219)
Supplement: Supplementary file 1 [file genes-12-00219-s001.pdf]

genes

MDPI

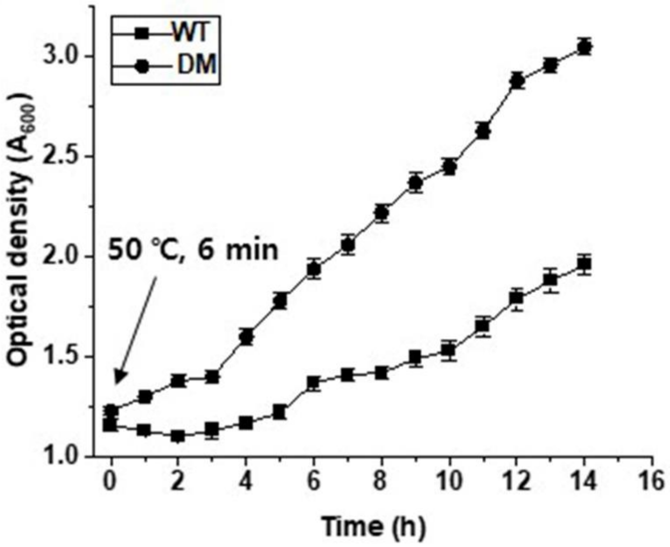

Figure S1. Cellular response to heat shock in *DaMDHAR*-expressing yeast cells. Log phase yeast cells exposed to heat shock at 50℃ for 6 min were harvested by centrifugation and resuspended into a fresh YPD broth medium. Optical density was measured at 600 nm in 1 h-intervals for 14 h. WT, wild-type cells with empty vector; DM, *DaMDHAR*-expressing transgenic cells.

Table S1. Genotype of *S. cerevisiae* strains used in this study.

Strain	Genotype	Source
BY4741	*MATa; his3Δ1; leu2Δ0; met15Δ0; ura3Δ0*	Euroscarf
WT	*MATa; his3Δ1; leu2Δ0; met15Δ0; ura3Δ0*; p426GPD	This study
DM	*MATa; his3Δ1; leu2Δ0; met15Δ0; ura3Δ0*; p426GPD::DaMDHAR	This study
A2 (*ara2Δ*)	*MATa; his3Δ1; leu2Δ0; met15Δ0; ura3Δ0; YMR041c::kanMX4*	Euroscarf
DA	*MATa; his3Δ1; leu2Δ0; met15Δ0; ura3Δ0; YMR041c::kanMX4*; p426GPD::DaMDHAR	This study

Table S2. Twofold changed genes in *DaMDHAR*–expressing yeast cells to FT stress.

Gene symbol	Fold change (DM /WT)	Gene	Description
YGL158W	16.716	RCK1	Protein kinase involved in oxidative stress response
YJL136W-A	13.785		Hypothetical protein
YIL134C-A	11.969		Hypothetical protein
tD(GUC)Q	11.343		Mitochondrial aspartate tRNA (tRNA-Asp)
YGR043C	10.059	NQM1	Transaldolase of unknown function
YJR038C	10.056		Dubious open reading frame
YGR052W	7.928	FMP48	Hypothetical protein
YER172C-A	7.322		Dubious open reading frame
YLR124W	7.322		Dubious open reading frame
YDR034W-B	6.267		Predicted tail-anchored plasma membrane protein
YJL195C	5.959		Dubious open reading frame
YBR190W	5.624		Dubious open reading frame
YLR047C	5.285	FRE8	Protein with sequence similarity to iron/copper reductases
YMR251W	5.008	GTO3	Omega class glutathione transferase

YOR374W	4.982	ALD4	Mitochondrial aldehyde dehydrogenase
YOR345C	4.970		Dubious open reading frame
YPL061W	4.828	ALD6	Cytosolic aldehyde dehydrogenase
YBR064W	4.597		Dubious open reading frame
YOR100C	4.597	CRC1	Mitochondrial inner membrane carnitine transporter
YIR021W-A	4.498		Hypothetical protein
YJL150W	4.248		Dubious open reading frame
YOR348C	3.630	PUT4	Proline permease
YLR364C-A	3.620		Dubious open reading frame
YDL024C	3.591	DIA3	Hypothetical protein
YFL056C	3.591		Putative aryl-alcohol dehydrogenase
YDR102C	3.569		Hypothetical protein
YGR161C	3.557	RTS3	Putative component of the protein phosphatase type 2A complex
YLR365W	3.551		Hypothetical protein
YBR200W-A	3.535		Hypothetical protein
YDL037C	3.535	BSC1	Protein of unconfirmed function
YBR141W-A	3.530		Dubious open reading frame
YOR028C	3.522	CIN5	Basic leucine zipper (bZIP) transcription factor of the yAP-1 family
YLR366W	3.502		Dubious open reading frame
YDR187C	3.385		Dubious open reading frame
YBR116C	3.364		Dubious open reading frame
YAL061W	3.349	BDH2	Putative medium-chain alcohol dehydrogenase with similarity to BDH1
YFL014W	3.227	HSP12	Plasma membrane protein involved in maintaining membrane organization
YJL108C	3.180	PRM10	Pheromone-regulated protein
YKL001C	3.169	MET14	Adenylylsulfate kinase
YGR088W	3.076	CTT1	Cytosolic catalase T
YOL024W	3.018		Hypothetical protein to have thiol-disulfide oxidoreductase active site
YBL074C	3.015	AAR2	Component of the U5 snRNP complex
YBR285W	2.993		Hypothetical protein
YIL101C	2.973	XBP1	Transcriptional repressor
YMR175W-A	2.966		Hypothetical protein
YMR175W	2.951	SIP18	Phospholipid-binding hydrophilin
YML054C	2.938	CYB2	Cytochrome b2 (L-lactate cytochrome-c oxidoreductase)
YLR282C	2.899		Dubious open reading frame
YML123C	2.860	PHO84	High-affinity inorganic phosphate (Pi) transporter
YNL276C	2.855		Dubious open reading frame
YCL073C	2.855	GEX1	Proton:glutathione antiporter
YPR039W	2.855		Dubious open reading frame
YNL277W	2.829	MET2	L-homoserine-O-acetyltransferase
YER175W-A	2.790		Hypothetical protein
YGR107W	2.790		Dubious open reading frame
YNL195C	2.767		Hypothetical protein; shares a promoter with YNL194C
YKL202W	2.765		Dubious open reading frame
YMR169C	2.758	ALD3	Cytoplasmic aldehyde dehydrogenase
YOL165C	2.753	AAD15	Putative aryl-alcohol dehydrogenase
YDL222C	2.670	FMP45	Integral membrane protein localized to mitochondria
YMR016C	2.648	SOK2	Nuclear protein that negatively regulates pseudohyphal differentiation
YPR065W	2.647	ROX1	Heme-dependent repressor of hypoxic genes

YGR161W-C	2.622		Hypothetical protein
YKL201C	2.611	MNN4	Putative positive regulator of mannosylphosphate transferase Mnn6
YAR069C	2.609		Dubious open reading frame
YAR070C	2.609		Dubious open reading frame
YBR296C	2.609	PHO89	Plasma membrane Na+/Pi cotransporter
YDL242W	2.609		Hypothetical protein
YKR069W	2.557	MET1	S-adenosyl-L-methionine uroporphyrinogen III transmethylase
YIL059C	2.527		Dubious open reading frame
YCR101C	2.517		Hypothetical protein
tD(GUC)M	2.517		Aspartate tRNA (tRNA-Asp)
YER062C	2.511	GPP2	DL-glycerol-3-phosphate phosphatase involved in glycerol biosynthesis
YPR160C-A	2.507		Dubious open reading frame
YDL210W	2.506	UGA4	GABA (gamma-aminobutyrate) permease
YKL096W	2.496	CWP1	Cell wall mannoprotein that localizes to birth scars of daughter cells
YKR104W	2.476		Putative transporter of the MRP subfamily
YLL061W	2.455	MMP1	High-affinity S-methylmethionine permease
YDR070C	2.435	FMP16	Hypothetical protein
YLL047W	2.434		Dubious open reading frame
YER121W	2.407		Hypothetical protein
YAL048C	2.403	GEM1	Outer mitochondrial membrane GTPase
YDL085W	2.403	NDE2	Mitochondrial external NADH dehydrogenase
YBR157C	2.387	ICS2	Hypothetical protein
YGR236C	2.385	SPG1	Protein required for high temperature survival during stationary phase
YNR014W	2.369		Hypothetical protein
YIL058W	2.364		Dubious open reading frame
YKL131W	2.361		Dubious open reading frame
YDR406W	2.343	PDR15	Plasma membrane ATP binding cassette (ABC) transporter
YGR242W	2.334		Dubious open reading frame
YKL106C-A	2.334		Hypothetical protein
YLR458W	2.334		Dubious open reading frame
YKR052C	2.330	MRS4	Iron transporter of the mitochondrial carrier family
YNL036W	2.322	NCE103	Carbonic anhydrase
YPR077C	2.317		Dubious open reading frame
YGR256W	2.314	GND2	6-phosphogluconate dehydrogenase (decarboxylating)
YPL222W	2.304	FMP40	Hypothetical protein
tG(GCC)M	2.284		Glycine tRNA (tRNA-Gly)
YER150W	2.237	SPI1	GPI-anchored cell wall protein involved in weak acid resistance
YGR049W	2.235	SCM4	Mitochondrial outer membrane hypothetical protein
YOR071C	2.223	NRT1	High-affinity nicotinamide riboside transporter
YLR164W	2.218	SHH4	Putative alternate subunit of succinate dehydrogenase (SDH)
YLL038C	2.214	ENT4	Hypothetical protein
tI(AAU)P2	2.214		Isoleucine tRNA (tRNA-Ile)
YDR384C	2.207	ATO3	Plasma membrane protein, putative ammonium transporter
YKL163W	2.194	PIR3	O-glycosylated covalently-bound cell wall protein
YIL055C	2.193		Hypothetical protein
YOR161C	2.184	PNS1	Hypothetical protein
YJL133C-A	2.183		Delta-Psi dependent mitochondrial Import protein of 8 kDa
YOL084W	2.173	PHM7	Hypothetical protein
YJR010W	2.171	MET3	ATP sulfurylase

YKL177W	2.170		Dubious open reading frame
YEL072W	2.170	RMD6	Protein required for sporulation
YGL230C	2.169		Hypothetical protein
YGR121C	2.159	MEP1	Ammonium permease
YKR039W	2.155	GAP1	General amino acid permease
YHR105W	2.149	YPT35	Endosomal hypothetical protein
YLR108C	2.146		Hypothetical protein
YDL183C	2.141		Protein that may form an active mitochondrial KHE system
YGL184C	2.138	STR3	Peroxisomal cystathionine beta-lyase
YBR117C	2.134	TKL2	Transketolase
YER054C	2.131	GIP2	Putative regulatory subunit of protein phosphatase Glc7p
YDR534C	2.115	FIT1	Mannoprotein that is incorporated into the cell wall
YOL059W	2.110	GPD2	NAD-dependent glycerol 3-phosphate dehydrogenase
YNL277W-A	2.106		Hypothetical protein
tT(AGU)O2	2.106		Threonine tRNA (tRNA-Thr)
YKL093W	2.103	MBR1	Protein involved in mitochondrial functions and stress response
YPL222C-A	2.101		Hypothetical protein
YOR339C	2.100	UBC11	Ubiquitin-conjugating enzyme
YPL135W	2.096	ISU1	Conserved protein of the mitochondrial matrix
YGL188C-A	2.090		Hypothetical protein
YMR182W-A	2.089		Mitochondrial MINi protein of 3 kDa
YMR262W	2.085		Hypothetical protein
YPR038W	2.085		Dubious open reading frame
YMR011W	2.083	HXT2	High-affinity glucose transporter of the major facilitator superfamily
YNL210W	2.082	MER1	mRNA-binding protein required for meiosis-specific mRNA splicing
YML128C	2.082	MSC1	Hypothetical protein
YOL060C	2.074	MAM3	Protein required for normal mitochondrial morphology
YPR167C	2.069	MET16	3'-phosphoadenylsulfate reductase
YKL062W	2.061	MSN4	Stress-responsive transcriptional activator
YHR015W	2.050	MIP6	Putative RNA-binding protein
YGR243W	2.050	MPC3	Highly conserved subunit of the mitochondrial pyruvate carrier (MPC)
YHR193C-A	2.035		Dubious open reading frame
YGR055W	2.034	MUP1	High affinity methionine permease
YJL213W	2.033		Hypothetical protein that may interact with ribosomes
YFR030W	2.028	MET10	Subunit alpha of assimilatory sulfite reductase;
YLR161W	2.022		Hypothetical protein
YPL136W	2.019		Dubious open reading frame
YIL167W	2.018	SDL1	Blocked reading frame otherwise encoding L-serine dehydratase
YPR196W	2.018		Putative maltose-responsive transcription factor
YOL109W	0.500	ZEO1	Peripheral membrane protein of the plasma membrane
YHR139C-A	0.499		Hypothetical protein
YMR149W	0.499	SWP1	Delta subunit of the oligosaccharyl transferase glycoprotein complex
YGL209W	0.498	MIG2	Zinc finger transcriptional repressor
YJR016C	0.497	ILV3	Dihydroxyacid dehydratase
YBR018C	0.497	GAL7	Galactose-1-phosphate uridyl transferase
tQ(UUG)H	0.496		Glutamine tRNA (tRNA-Gln)
YDR241W	0.494	BUD26	BUD site selection
YBR182C-A	0.494		Hypothetical protein
YEL067C	0.494		Hypothetical protein
YPR197C	0.494		Dubious open reading frame

tX(XXX)D	0.492		tRNA of undetermined specificity
tK(CUU)G1	0.488		Lysine tRNA (tRNA-Lys)
YGR087C	0.487	PDC6	Minor isoform of pyruvate decarboxylase
YHR199C-A	0.487	NBL1	Subunit of the conserved chromosomal passenger complex (CPC)
YNR074C	0.485	AIF1	Mitochondrial cell death effector
tM(CAU)J2	0.484		Methionine tRNA (tRNA-Met)
YMR104C	0.484	YPK2	Protein kinase similar to S/T protein kinase Ypk1p
tK(CUU)G2	0.481		Lysine tRNA (tRNA-Lys)
YNL190W	0.480		Hydrophilin essential in desiccation-rehydration process
YLR406C-A	0.478		Hypothetical protein
YBR298C	0.476	MAL31	Maltose permease; high-affinity maltose transporter
YFL012W	0.476		Hypothetical protein
tR(UCU)J2	0.476		Arginine tRNA (tRNA-Arg)
YER066C-A	0.474		Dubious open reading frame
YMR122C	0.473		Hypothetical protein
YMR122W-A	0.472		Hypothetical protein
YDL247W	0.472	MPH2	Alpha-glucoside permease
tE(UUC)G2	0.470		Glutamate tRNA (tRNA-Glu)
YFR027W	0.468	ECO1	Acetyltransferase
RNA170	0.468		RNA polymerase III transcribed RNA of unknown function
tK(CUU)I	0.464		Lysine tRNA (tRNA-Lys)
YFR055W	0.462	IRC7	Beta-lyase involved in the production of thiols
YDR345C	0.459	HXT3	Low affinity glucose transporter of the major facilitator superfamily
YGR249W	0.454	MGA1	Protein similar to heat shock transcription factor
YER147C-A	0.454		Dubious open reading frame
YKR077W	0.453	MSA2	Putative transcriptional activator
YGR139W	0.447		Dubious open reading frame
YER106W	0.446	MAM1	Monopolin
YOR180C	0.441	DCI1	Peroxisomal delta(3,5)-delta(2,4)-dienoyl-CoA isomerase protein
tK(UUU)G2	0.440		Lysine tRNA (tRNA-Lys)
YHR182C-A	0.439		Dubious open reading frame
tR(CCU)J	0.439		Arginine tRNA (tRNA-Arg)
YCR006C	0.436		Hypothetical protein
YDR136C	0.436	VPS61	Vacuolar protein sorting
YLR402W	0.436		Dubious open reading frame
YPL126W	0.434	NAN1	U3 snoRNP protein
YGL063C-A	0.425		Dubious open reading frame
YCR083W	0.418	TRX3	Mitochondrial thioredoxin
YDR537C	0.418		Dubious open reading frame
YKR075C	0.417		Hypothetical protein similar to Reg1p
YKL124W	0.415	SSH4	Specificity factor required for Rsp5p-dependent ubiquitination
YAR068W	0.412		Fungal-specific hypothetical protein
YNL103W-A	0.412		Dubious open reading frame
snR191	0.404		H/ACA box small nucleolar RNA (snoRNA)
YHL037C	0.395		Hypothetical protein
YDL236W	0.392	PHO13	Conserved phosphatase acting as a metabolite repair enzyme
YFR008W	0.391	FAR7	Protein involved in recovery from pheromone-induced cell cycle arrest
tT(AGU)J	0.387		Threonine tRNA (tRNA-Thr)
YHR185C	0.384	PFS1	Sporulation protein required for prospore membrane formation
Q0250	0.371	COX2	subunit II of cytochrome c oxidase (Complex IV)

YML084W	0.353		Dubious open reading frame
YER135C	0.348		Hypothetical protein
YJR128W	0.348		Hypothetical protein
YKR005C	0.346		Hypothetical protein
YEL035C	0.330	UTR5	Hypothetical protein
YOR135C	0.322		Dubious open reading frame
YKR075W-A	0.317		Dubious open reading frame
YAR060C	0.311		Dubious open reading frame
YEL074W	0.297		Dubious open reading frame
tI(AAU)L2	0.295		Isoleucine tRNA (tRNA-Ile)
YNR069C	0.263	BSC5	Hypothetical protein
YBR232C	0.260		Dubious open reading frame
YHL046W-A	0.232		Dubious open reading frame
YER067W	0.232	RGI1	Hypothetical protein involved
YER067C-A	0.219		Dubious open reading frame
YNR072W	0.217	HXT17	Putative transmembrane polyol transporter
YKL102C	0.209		Hypothetical protein
YML054C-A	0.165		Hypothetical protein
YDL026W	0.154		Dubious open reading frame
YER180C-A	0.138	SLO1	Protein interacting with Arl3p
YOR073W-A	0.135		Dubious open reading frame
YHR069C-A	0.115		Dubious open reading frame
YJL197C-A	0.115		Dubious open reading frame
YCR021C	0.110	HSP30	Negative regulator of the H(+)-ATPase Pma1p

Table S3. GO–based classification of upregulated genes in *DaMDHAR*–expressing yeast cells.

GO ID	GO ontology	Ontology source	Associated genes
GO:0044459	Plasma membrane part	CC	GAP1, HXT2, MEP1, MMP1, MUP1, PHO84, PNS1, PUT4, UGA4, YDR034W-B
GO:0006811	Ion transport	BP	ATO3, FIT1, GAP1, GEX1, HXT2, MEP1, MMP1, MPC3, MRS4, MUP1, PHM7, PHO84, PUT4, UGA4, YDL183C
GO:0015291	Secondary active transmem-brane transporter activity	MF	GAP1, GEX1, HXT2, MMP1, MUP1, PHO84, PUT4, UGA4
GO:0005887	Integral component of plasma membrane	CC	GAP1, HXT2, MEP1, MMP1, MUP1, PHO84, PNS1, PUT4, UGA4
GO:0006820	Anion transport	BP	ATO3, GAP1, MMP1, MPC3, MUP1, PHO84, PUT4, UGA4
GO:0008324	cation transmembrane Trans-porter activity	MF	ATO3, GEX1, HXT2, MEP1, MRS4, PHO84, PNS1, UGA4
GO:0015297	Antiporter activity	MF	GAP1, GEX1, MMP1, MUP1, PUT4, UGA4
GO:0098656	Anion transmembrane Transport	BP	ATO3, MPC3, PHO84
GO:0046942	Carboxylic acid transport	BP	ATO3, GAP1, MMP1, MPC3, MUP1, PUT4, UGA4
GO:0015807	L-amino acid transport	BP	MMP1, MUP1, UGA4
GO:0003333	Amino acid transmembrane transport	BP	GAP1, MMP1, MUP1, PUT4, UGA4

GO:0015175	Neutral amino acid transmembrane transporter activity	MF	GAP1, MUP1, PUT4
GO:0015179	L-amino acid transmembrane transporter activity	MF	GAP1, MMP1, MUP1, PUT4, UGA4
GO:0015295	Solute:proton symporter activity	MF	HXT2, PHO84, UGA4
GO:0046873	Metal ion transmembrane transporter activity	MF	GEX1, MRS4, PHO84, UGA4
GO:1902475	L-alpha-amino acid transmembrane transport	BP	MMP1, MUP1, UGA4
GO:0044459	Plasma membrane part	CC	GAP1, HXT2, MEP1, MMP1, MUP1, PHO84, PNS1, PUT4, UGA4, YDR034W-B
GO:0006811	Ion transport	BP	ATO3, FIT1, GAP1, GEX1, HXT2, MEP1, MMP1, MPC3, MRS4, MUP1, PHM7, PHO84, PUT4, UGA4, YDL183C
GO:0015291	Secondary active transmembrane transporter activity	MF	GAP1, GEX1, HXT2, MMP1, MUP1, PHO84, PUT4, UGA4
GO:0005887	Integral component of plasma membrane	CC	GAP1, HXT2, MEP1, MMP1, MUP1, PHO84, PNS1, PUT4, UGA4
GO:0008324	Cation transmembrane transporter activity	MF	ATO3, GEX1, HXT2, MEP1, MRS4, PHO84, PNS1, UGA4
GO:0008519	Ammonium transmembrane transporter activity	MF	ATO3, MEP1, PNS1
GO:0015295	Solute:proton symporter activity	MF	HXT2, PHO84, UGA4
GO:0046873	Metal ion transmembrane transporter activity	MF	GEX1, MRS4, PHO84, UGA4
GO:0032592	Integral component of mitochondrial membrane	CC	GEM1, MPC3, YDL183C
GO:0055076	Transition metal ion homeostasis	BP	FIT1, GEX1, ISU1, MAM3
GO:0000103	Sulfate assimilation	BP	MET1, MET10, MET14, MET16, MET3
GO:0019379	Sulfate assimilation, phosphoadenylyl sulfate reduction by phosphoadenylyl-sulfate reductase (thioredoxin)	BP	MET14, MET16, MET3
GO:0070814	Hydrogen sulfide biosynthetic process	BP	MET10, MET14, MET16, MET3
GO:0009066	Aspartate family amino acid metabolic process	BP	MET1, MET10, MET14, MET16, MET2, MET3, SOK2, STR3, XBP1
GO:0009069	Serine family amino acid metabolic process	BP	MET10, MET14, MET16, MET2, MET3, STR3
GO:0046394	Carboxylic acid biosynthetic process	BP	ALD3, ALD4, ALD6, MET1, MET10, MET14, MET16, MET2, MET3, STR3
GO:0009086	Methionine biosynthetic process	BP	MET1, MET10, MET14, MET16, MET2, MET3, STR3
GO:0019344	Cysteine biosynthetic process	BP	MET10, MET14, MET16, MET3, STR3
GO:0006066	Alcohol metabolic process	BP	ALD4, ALD6, GPD2, GPP2, NDE2
GO:0006732	Coenzyme metabolic process	BP	ALD3, ALD4, ALD6, GND2, GPD2, NDE2, NQM1, TKL2

GO:0004029	Aldehyde dehydrogenase (NAD) activity	BP	ALD3, ALD4, ALD6
GO:0006067	Ethanol metabolic process	BP	ALD4, ALD6, NDE2
GO:0072330	Monocarboxylic acid biosynthetic process	BP	ALD3, ALD4, ALD6
GO:0046496	Nicotinamide nucleotide metabolic process	BP	ALD4, ALD6, GND2, GPD2, NDE2, NQM1, TKL2
GO:0006098	Pentose-phosphate shunt	BP	GND2, NQM1, TKL2
GO:0006739	NADP metabolic process	BP	ALD4, ALD6, GND2, NQM1, TKL2

*BP, biological process; CC, cellular components; MF, molecular function.

Table S4. Kyoto Encyclopedia of Genes and Genomes (KEGG)–based classification of upregulated genes in *DaMDHAR*–expressing yeast cells.

GOID	GO Term	Associated genes
GO:0000030	Pentose phosphate pathway	GND2, NQM1, TKL2
GO:0000010	Glycolysis/gluconeogenesis	ALD3, ALD4, ALD6
GO:0000340	Histidine metabolism	ALD3, ALD4, ALD6
GO:0000380	Tryptophan metabolism	ALD4, ALD6, CTT1
GO:0000410	Beta-alanine metabolism	ALD3, ALD4, ALD6
GO:0000561	Glycerolipid metabolism	ALD4, ALD6, GPP2
GO:0000620	Pyruvate metabolism	ALD4, ALD6, CYB2
GO:0000920	Sulfur metabolism	MET10, MET14, MET16, MET3

Table S5. GO–based classification of downregulated genes in DaMDHAR–expressing yeast cells.

GO ID	GO Term	Ontology source	Associated genes
GO:0016668	Oxidoreductase activity, acting on a sulfur group of donors, NAD(P) as acceptor	MF	AIF1, TRX3
GO:0022904	Respiratory electron transport chain	BP	AIF1, COX2
GO:0015151	Alpha-glucoside transmembrane transporter activity	MF	MAL31, MPH2
GO:0000023	Maltose metabolic process	BP	MAL31, MPH2
GO:0005351	Sugar:proton symporter activity	MF	HXT3, MAL31, MPH2
GO:0000017	Alpha-glucoside transport	BP	MAL31, MPH2
GO:0005355	Glucose transmembrane transporter activity	MF	HXT3, MAL31, MPH2
GO:1904659	Glucose transmembrane transport	BP	HXT3, MAL31, MPH2
GO:0046323	Glucose import	BP	HXT3, MAL31, MPH2

Table S6. KEGG–based classification of downregulated genes in *DaMDHAR*–expressing yeast cells.

GO ID	GO Term	Associated genes
GO:0000010	Glycolysis/gluconeogenesis	PDC6
GO:0000190	Oxidative phosphorylation	COX2
GO:0000510	N-glycan biosynthesis	SWP1
GO:0000513	Various types of N-glycan biosynthesis	SWP1
GO:0004141	Protein processing in endoplasmic reticulum	SWP1
GO:0000270	Cysteine and methionine metabolism	IRC7
GO:0000450	Selenocompound metabolism	IRC7
GO:0000290	Valine, leucine and isoleucine biosynthesis	ILV3
GO:0000770	Pantothenate and CoA biosynthesis	ILV3
GO:0003008	Ribosome biogenesis in eukaryotes	NAN1
GO:0004113	Meiosis	HXT3, MAM1
